# Organocatalytic Thioesterification of a Conjugated α,β-Unsaturated Dialdehyde

**DOI:** 10.3390/ijms27135941

**Published:** 2026-07-01

**Authors:** Kamil Hanek, Kacper Grzegorczyk, Michał Dutkiewicz, Patrycja Żak

**Affiliations:** 1 Faculty of Chemistry, Adam Mickiewicz University in Poznań, Uniwersytetu Poznańskiego St. 8, 61-614 Poznań, Poland; kamhan1@amu.edu.pl (K.H.); kacgrz4@st.amu.edu.pl (K.G.); 2 Poznan Science and Technology Park, Adam Mickiewicz University Foundation, Rubiez St. 46, 61-612 Poznań, Poland; michal.dutkiewicz@ppnt.poznan.pl

**Keywords:** thioester, acrolein, sulfa-Michael adduct, *N*-heterocyclic carbene, organocatalysis

## Abstract

Thioesterification of (2*E*,2′*E*)-3,3′-(1,4-phenylene)diacrylaldehyde with thiols has been performed using a bulky *N*-heterocyclic carbene (NHC) as an organocatalyst. This metal-free protocol enables efficient synthesis of new mono and difunctional acrolein derivatives in high isolated yields under mild conditions. Importantly, the reaction proceeds in the absence of external additives or oxidants, and requires only low organocatalyst loadings, thereby facilitating the straightforward isolation of structurally defined sulfur-containing acrolein derivatives in both symmetric and asymmetric forms. The resulting functional acrolein derivatives are the hitherto underexplored class of functional building blocks with promising potential for biomedical materials, including drug delivery systems and other biomaterials applications.

## 1. Introduction

The α,β-unsaturated carbonyl compounds represent an important class of bioactive structural motifs that are widely found in natural products and synthetic systems of biomedical relevance. Their well-defined electrophilic properties and controllable reactivity profiles make them highly suitable for applications in medicinal chemistry, chemical biology and biomaterials research. A broad range of α,β-unsaturated carbonyl derivatives have been used as functional components of many cosmetics [1] and pharmaceuticals [2], owing to their broad spectrum of biological activities, including antiviral [3,4,5], anti-inflammatory [6], antibacterial [7,8,9,10], antioxidative [11,12], anticancer [13,14], antidiabetic [15], antifungal [16], and antimalarial [17] effects. From a biomaterials perspective, the biomedical significance of α,β-unsaturated carbonyl compounds is not limited to their inherent bioactivity but also arises from their ability to modulate biological responses through covalent or reversible interactions with biomolecular targets. Notably, several members of this class have been shown to inhibit proteasomal activity [18] and to induce anti-proliferative and pro-apoptotic responses in cancer cells [18,19], making them valuable candidates for incorporation into anticancer delivery platforms. Their ability to interfere with mitochondrial function further contributes to antineoplastic efficacy [20]. Beyond oncology-related applications, α,β-unsaturated carbonyl derivatives can act as immunosuppressive agents that abolish the overactive immune system in autoimmune disease and augmentation agents that enhance the immunological response during viral infections [21]. Moreover, functionalized carbonyl derivatives have been reported to increase the efficacy of vaccines and improve the pharmacokinetic profiles of therapeutic agents, as demonstrated by curcumin-based delivery systems [21,22].

Despite many advantages, α,β-unsaturated carbonyl compounds are well known for their pronounced toxicity toward both environmental and biological systems. This reactivity is primarily associated with their ability to act as Michael acceptors, enabling covalent modification of nucleophilic biomolecules such as cysteine and selenocysteine residues in proteins, enzymes, and DNA [23,24,25]. As a consequence, exposure to these electrophiles may induce electrophilic stress, oxidative damage, and disruption of cellular redox homeostasis [23,24,25]. Among these compounds, acrolein and related acrylates are particularly well studied and have been implicated in a range of pathological processes, including sensory deficits, spinal cord injury, and broader neurotoxicity associated with neurodegenerative disorders [25,26,27]. In addition, endogenous formation of reactive aldehydes such as acrolein through lipid peroxidation has been recognized as a key contributor to oxidative stress-mediated cellular damage [27,28]. Recent studies further suggest their potential involvement in ferroptosis, an iron-dependent form of regulated cell death driven by lipid peroxidation processes [29,30]. Notably, although acrolein also contributes to antimicrobial activity in certain biological contexts, its high cytotoxicity and lack of selectivity significantly limit its direct biomedical applicability [31]. Therefore, strategies aimed at modulating or attenuating the intrinsic reactivity of these aldehydes while preserving their beneficial properties have attracted considerable attention. Various detoxification and functionalization approaches have been reported, including iron-mediated reduction processes [32] and the use of aldehyde scavengers for removal from biologically relevant systems [33]. From a synthetic perspective, one of the most common approaches for carbonyl functionalization involves nucleophilic addition reactions, including thioesterification. Traditionally, thioesters are synthesized by nucleophilic substitution of the carbonyl group of acyl chlorides, carboxylic acids or acid anhydrides with thiols or disulfides [34], or through transition-metal-catalyzed methods [35,36,37,38,39,40]. Unfortunately, all the above-mentioned methodologies suffer from notable drawbacks, including moderate efficiency, harsh reaction conditions, mandatory use of oxidants, long reaction time, and the use of toxic solvents [35,36,37,38,39,40]. These challenges continue to motivate the development of more efficient and sustainable approaches for thioester formation.

Organocatalysis offers a great alternative to thioester synthesis, as these derivatives can be synthesized through *N*-heterocyclic carbenes (NHC) catalysis. However, effective suppression of common limitations—such as elevated reaction temperatures and high catalyst loadings—often requires the use of bulky NHC catalysts [41,42,43]. This type of organocatalysts, featuring pronounced steric hindrance around the nitrogen atoms, can overcome many of these limitations by enabling highly chemoselective transformations under mild conditions with excellent efficiency and near-quantitative atom economy. Our research group has previously reported the successful application of bulky NHCs in the functionalization of α,β-unsaturated carbonyl compounds with a range of nucleophiles, yielding products of relevance to biologically oriented molecular design [44,45,46,47,48]. Building on these findings, we identified the derivatization of acroleins as a critical next step toward assessment of the further perspectives and possible limitations of bulky NHC-mediated organocatalysis.

Herein, we report a new organocatalytic strategy for the functionalization of *p*-phenylene-bridged dienal with a range of thiols, leading to a novel class of symmetric and unsymmetric bis-substituted sulfur-containing products. The presented methodology provides access to structurally diverse α,β-unsaturated carbonyl derivatives, which could be explored in the future for the development of functional biomolecules and bioactive materials.

## 2. Results and Discussion

### 2.1. Design and Optimization of the Reaction System

**Figure 1:**
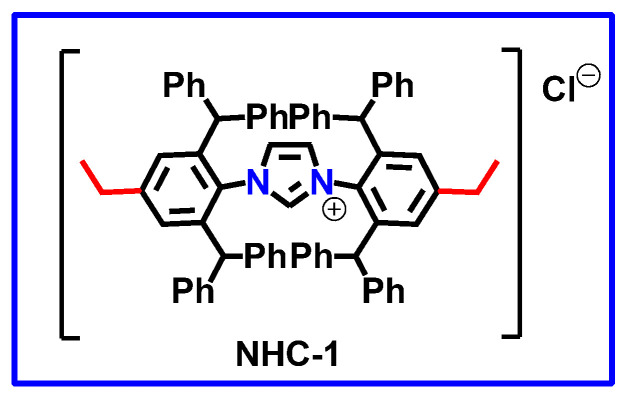
Structure of organocatalyst precursor **NHC-1**.

At the initial stage of the study, a series of catalytic experiments was conducted to determine the optimal conditions for the functionalization of (2*E*,2′*E*)-3,3′-(1,4-phenylene)diacrylaldehyde (**1a**). 4-Methoxythiophenol (**2a**) was chosen as the model reaction partner owing to its low cost and high commercial availability. The process was catalyzed by the NHC salt (**NHC-1**) containing sterically crowded substituents at the terminal nitrogen atoms of the imidazole ring, synthesized according to the procedure reported by Delaude’s group [45] (Figure 1). Based on our previous work, smaller NHCs lead to unsatisfactory results in the thioesterification process, and hence the catalysts comparison was omitted in this study [40].

The addition of reagents (**1a**, **2a**) to an acetone solution containing 20 mol% of **NHC-1** and 20 mol% of KHMDS, at 60 °C, resulted in a complete conversion of the substrates after 24 h. The ^1^H NMR analysis of the crude reaction mixture revealed the selective formation of the expected symmetrical bis-thioesterification product **P1** in a quantitative yield (Table 1, Entry 1). Subsequently, **NHC-1** loading was gradually decreased, and all reactions, except that with 2.5 mol% catalyst, achieved full conversion (Table 1, Entries 2–4). When the imidazolium salt concentration was lowered to 2.5 mol%, the conversion reached only 45% of both reagents (Table 1, Entry 5). The experiment carried out without **NHC-1** did not afford any products, even though the reaction time was extended to 72 h (Table 1, Entry 6). Having established the optimal catalyst loading, the effect of a base was investigated. From among the tested bases, KHMDS provided the most effective course of the reaction, as it ensured quantitative conversion of substrate **1a** and led selectively to product **P1** (Table 1, Entries 1–4). The process occurred selectively also in the presence of potassium carbonate, potassium *t*-butoxide and trimethylamine, but then the conversion of the substrates dropped down to 85%, 65% and 50%, respectively (Table 1, Entries 7–9). Control experiments confirmed that the presence of a base is essential for the reaction to proceed (Table 1, Entry 10). To gain further insight into its role, the reaction was carried out using the previously isolated carbene, which afforded high conversion (Table 1, Entry 11). Increasing the amount of the base to two equivalents had no noticeable effect on the reaction outcome (Table 1, Entry 12). These results indicate that the base serves only to generate the free carbene. As shown in Table 1, both the solvent and reaction temperature played a crucial role in determining the course of the reaction. The highest efficiency was obtained in acetone at 60 °C, while the use of *i*-PrOH, MBIK or EtOAc led to markedly lower substrate conversion (Table 1, Entry 4 vs. Entries 13–15). Lowering the temperature reduced the catalyst activity, affording 80% conversion at 40 °C and 65% at room temperature (Table 1, Entries 16–17). Further tests conducted under ambient atmosphere confirmed that an inert environment is required (Table 1, Entry 18). The final parameter optimized was the reaction time. The obtained results clearly confirmed that quantitative conversion of the substrates was achieved within only 15 min (Table 1, Entry 20 vs. Entry 21). A further decrease in reaction time led to unsatisfactory substrates conversions (Table 1, Entry 22). Overall, the conditions described in Entry 21 were determined to be optimal for this process.

In accordance with the standard practice of our laboratory, all catalytic tests were repeated three times and the obtained results indicated a high reproducibility of the method. To better verify the reproducibility of yields and reaction conditions, the model reaction was repeated five times. The standard deviation for the obtained yield values was calculated to be 1.52%. The results of the statistical analysis demonstrate good reproducibility and reliability of the described method (See ESI for details).

### 2.2. Scope of the Reaction

**Scheme 1:**
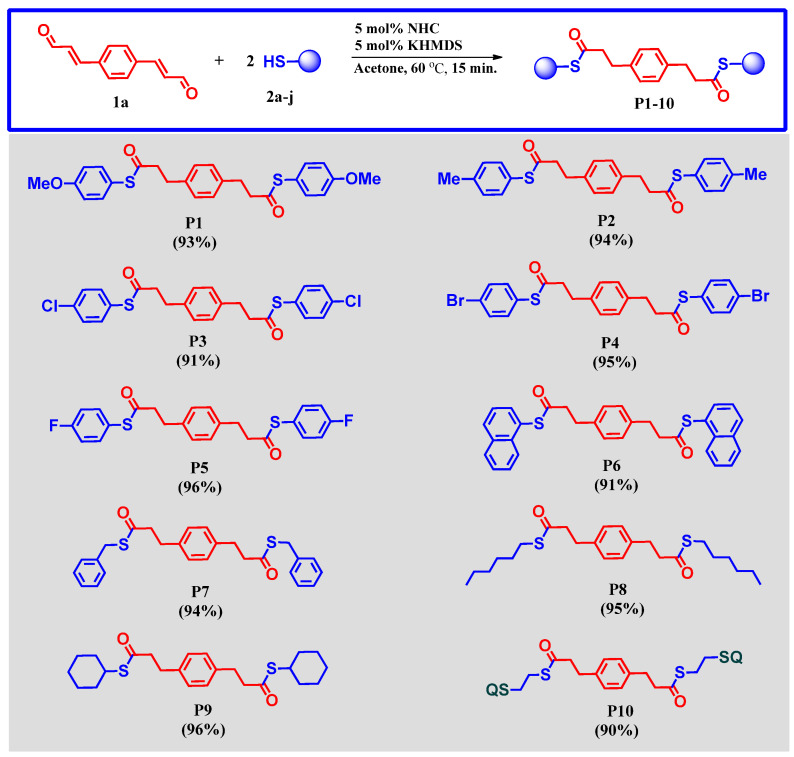
Functionalization of **1a** with **2a**–**j** (conditions: acetone, 60 °C, (**1a**):(**2a**–**j**) = 1:2, **NHC-1** = IPr*^OMe^·HCl, [**NHC-1**] = [KHMDS] = 5 mol%, argon). Isolated yields are given under the structures. **SQ**—mercaptopropylisobutylsilsesquioxyl group.

**Figure 2:**
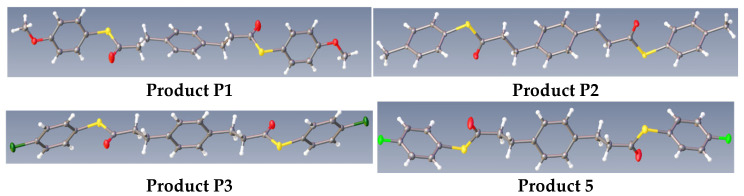
Perspective views of the products **P1**–**P3** and **P5**.

Following the identification of the optimal conditions, we investigated the scope of the designed organocatalytic system. The reactivity of **1a** was evaluated with a range of commercially available thiols (Scheme 1). As shown above, the developed protocol proved applicable to aryl (**2a**–**f**), alkylaryl (**2g**), and alkyl (**2h**–**i**) thiols. Interestingly, it was also effective for mercaptosubstituted silsesquioxane (**2j**). This compound was chosen because, to the best of our knowledge, there are no reports on the modification of acroleins with SQs. In addition, SQ-based materials exhibit unique properties that broaden the scope of their potential applications [20]. In all cases, nearly quantitative conversion of the substrates was achieved, affording the corresponding products in excellent isolated yields. No significant differences in the reaction efficiency were observed for aryl thiols bearing both electron-withdrawing and electron-donating substituents. Furthermore, no by-products were detected, indicating that the proposed method can be applied to a broad range of reagents. All products (**P1**–**P10**) were purified by column chromatography on silica gel using DCM or a 1:1 (*v*/*v*) mixture of *n*-hexane and DCM as eluents. In addition, single crystals suitable for X-ray diffraction were obtained for the products formed in the reactions of (2*E*,2′*E*)-3,3′-(1,4-phenylene)diacrylaldehyde (**1a**) with 4-methoxythiophenol (**2a**), 4-methylthiophenol (**2b**), 4-chlorothiophenol (**2c**), and 4-fluorothiophenol (**2e**) (Figure 2). The crystals were grown by dissolving **P1**–**P3** and **P5** in *n*-hexane/DCM mixture followed by slow evaporation of the solvents (See ESI for details).

**Scheme 2:**
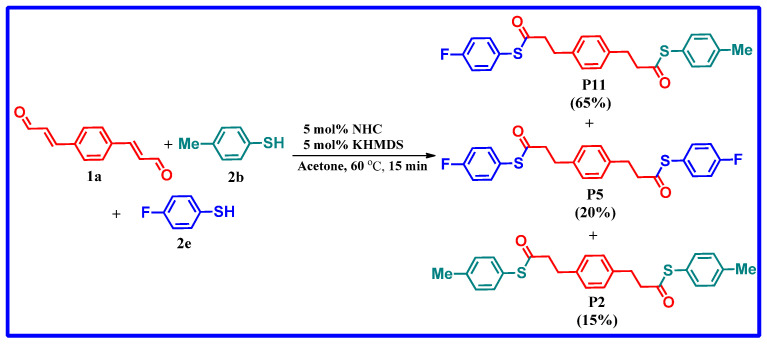
Functionalization of **1a** with **2b** and **2e** (conditions: acetone, 60 °C, (**1a**):(**2b**):(**2e**) = 1:1:1, **NHC-1** = IPr*^OMe^·HCl, [**NHC-1**] = [KHMDS] = 5 mol%, argon). Yields are given in parentheses.

After the synthesis of symmetrically functionalized bis(thioesters), we explored the possibility of obtaining unsymmetrical products through the reaction of **1a** with two different thiols (**2a**–**j**). Addition of 5.0 mol% of **NHC-1** to an equimolar solution of **1a**, 4-methylthiophenol (**2b**) and 4-fluorothiophenol (**2e**) in acetone, followed by stirring for 24 h, resulted in complete conversion of the substrates and the formation of a mixture of two symmetrical products (**P2**, **P5**) and an unsymmetrical one (**P11**) at the ratio of 20:15:65 (Scheme 2).

Because the thiols used in the synthesis of asymmetric products exhibit highly similar steric and electronic properties, the observed product distribution is not expected to arise from thermodynamic effects, but rather from statistical and minor kinetic factors. In a 1:1 dual-addition system, formation of the cross-product is statistically favored over either homodimer (2:1:1 baseline ratio), and the observed distribution (65:20:15) is consistent with this model, with a slight kinetic enhancement. This deviation may result from slightly different reactivity of the mono-functionalized intermediate formed after the first addition step, which can preferentially react with the second thiol rather than undergo self-coupling. These effects are commonly observed in sequential functionalization processes and reflect minor kinetic biases rather than thermodynamic control.

**Scheme 3:**
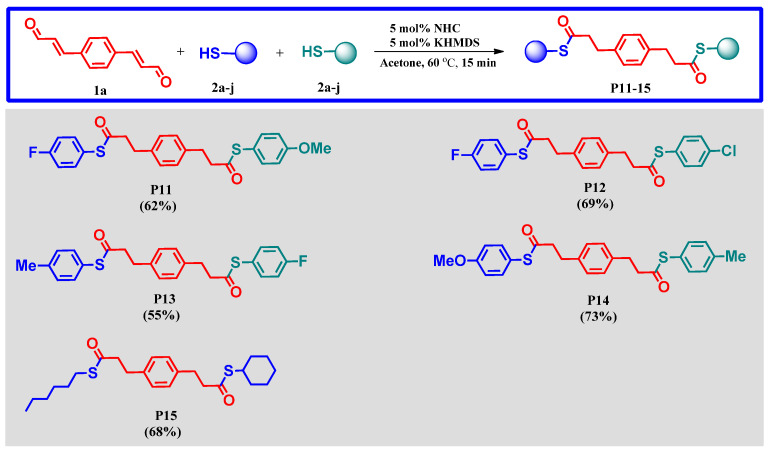
Functionalization of **1a** with two different thiols (**2a**–**j**) (conditions: acetone, 60 °C, (**1a**):(**2a**–**j**) = 1:1:1, **NHC-1** = IPr*^OMe^·HCl, [**NHC-1**] = [KHMDS] = 5 mol%, argon). Isolated yields are given in parentheses.

Despite the lack of complete selectivity, we decided to continue our investigations in this area, as the expected unsymmetrical substitution derivative was obtained as the major product and could be readily isolated from the reaction mixture. We also checked the possibility of first obtaining the mono-substituted product, followed by its modification through the addition of an equivalent of another thiol. However, this approach did not improve the selectivity of the process. Therefore, all subsequent experiments were carried out following the model reaction procedure, with all reagents introduced simultaneously into the reaction mixture. The results are summarized in Scheme 3. In all cases, we observed complete functionalization of the two internal C–C double bonds of *p*-phenylene-bridged dienal, with the expected unsymmetrical product formed in significant excess. All products were isolated by column chromatography and characterized using spectroscopic and mass spectrometric methods.

**Scheme 4:**
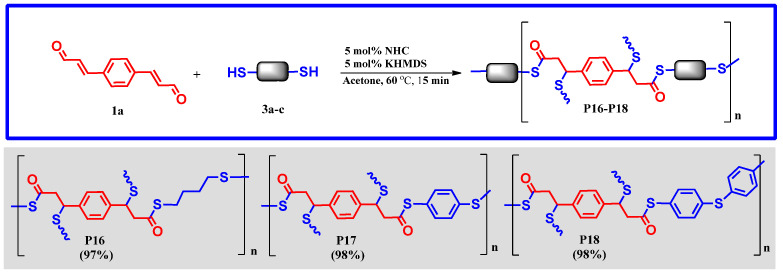
Functionalization of **1a** with **3a**–**c** (conditions: acetone, 60 °C, (**1a**):(**3a**–**c**) = 1:1, **NHC-1** = IPr*^OMe^·HCl, [**NHC-1**] = [KHMDS] = 5 mol%, argon). Isolated yields are given in parentheses.

The successful NHC-catalyzed reactions of **1a** with a series of thiols (**2a**–**j**) prompted us to extend the study to dithiols in order to evaluate the applicability of the developed methodology for the synthesis of polymeric materials. Relevant tests were performed using equimolar amounts of **1a** and 1,4-butanedithiol (**3a**), 1,4-benzenedithiol (**3b**), and 4,4′-thiobisbenzenethiol (**3c**) (Scheme 4).

In contrast to the previous experiments, precipitation was observed in all cases during the course of the reaction. The resulting products were insoluble, which prevented their analysis and structural confirmation using ^1^H and ^13^C NMR spectroscopies. For the same reason, GPC analysis, which is essential for polymer characterization, could not be performed to determine the molecular weight and molecular weight distribution of the obtained materials. Nevertheless, ATR FT-IR spectroscopy was employed to obtain structural information on the insoluble products, as this technique is suitable for the analysis of solid samples. The FT-IR spectra of samples **P16**–**P18** are presented in Figures S36–S38 (See ESI). Analysis of the spectra revealed a shift in the carbonyl (C=O) band maximum present in the spectrum of substrate **1a** from 1658 cm^−1^ toward higher wavenumbers (1700–1684 cm^−1^), the absence of the bands at 1617 and 974 cm^−1^ characteristic of alkenyl C=C bonds, and the absence of the 1420 cm^−1^ band characteristic of C-H vibrations of alkenyl groups present in the spectrum of substrate **1a**. In addition, no S-H stretching vibration bands, typically observed for the dithiols at approximately 2550 cm^−1^, were detected. At the same time, a new band with a maximum at 700 cm^−1^, characteristic of C-S vibrations in thioesters, was observed in the spectra of samples **P16**–**P18**. These observations are consistent with the occurrence of the polymerization process according to the proposed mechanism involving β-protonation of α,β-unsaturated enals [44]. However, the FT-IR spectra also revealed additional bands with maxima at approximately 1259 cm^−1^ and low-intensity bands at approximately 607 cm^−1^, attributable to C-S-C and C-S vibrations, respectively, suggesting the formation of thioether linkages via a competing thia-Michael addition pathway. Further evidence of reduced selectivity in the polythioesterification process when dithiols were employed is provided by additional carbonyl (C=O) bands centered at approximately 1780 cm^−1^ and broad absorptions in the 3700–3200 cm^−1^ region, characteristic of O–H stretching vibrations. These features may indicate the presence of water in the investigated samples and/or the occurrence of oxidative side reactions during the polymerization. Taken together, the FT-IR results suggest that both the β-protonation of α,β-unsaturated enals and a competing thia-Michael addition pathway may occur during polymer formation. The coexistence of these processes could account for the formation of crosslinked polymer networks and the resulting insolubility of the obtained materials. The lower selectivity observed in the polymerization reactions involving dithiols, compared to analogous reactions using monothiols, may arise from diffusion limitations and steric effects associated with the growing polymer chains. However, further studies are required to verify this hypothesis and to elucidate the structure of these materials in greater detail.

**Figure 3:**
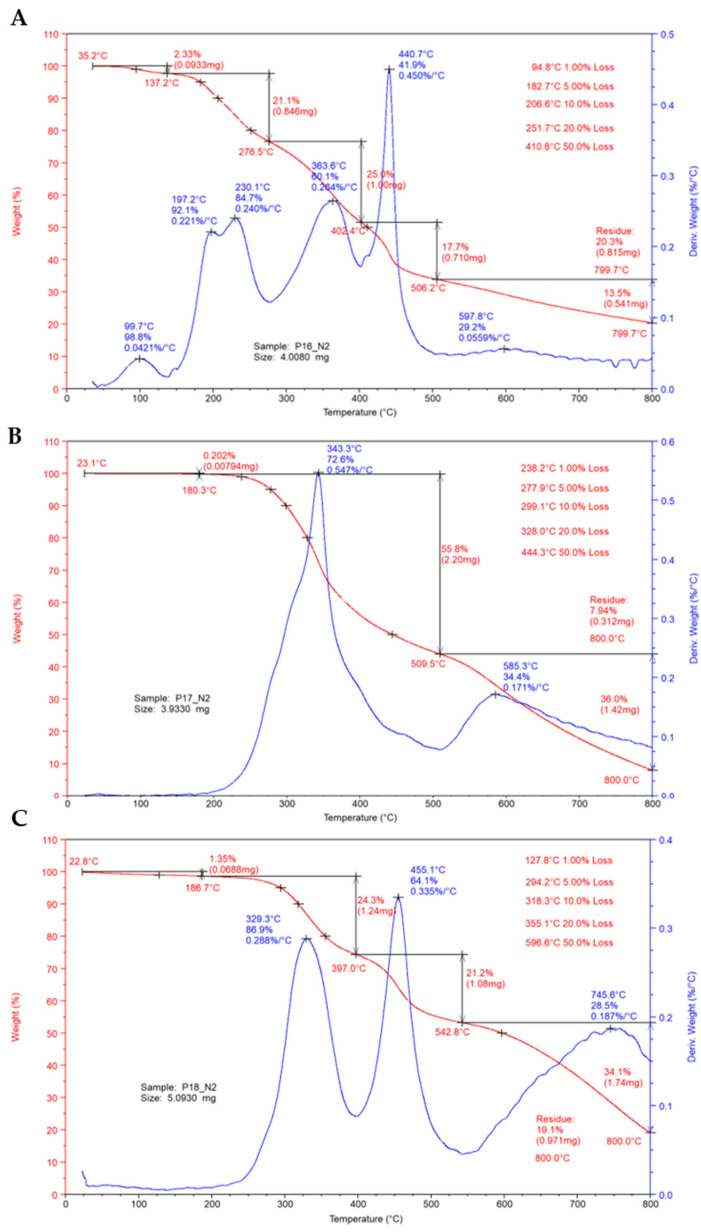
TG and DTG curves for products **P16** (**A**), **P17** (**B**) and **P18** (**C**).

To further characterize the obtained materials, samples **P16**–**P18** were subjected to thermogravimetric analysis. The TG and DTG curves recorded under a N_2_ atmosphere up to 800 °C are presented in Figure 3. The thermal decomposition process of the polythioesters obtained in the reaction of **1a** with dithiols **3a**–**c** strongly depends on the chemical structure of the segment derived from the dithiol. The general trend in the thermal stability of these polymers is an increase with increasing aromaticity and rigidity of the main chain.

Sample **P16**, containing a flexible aliphatic tetramethylene chain, exhibits the lowest thermal stability in the entire series. The presence of aliphatic C-H and C-C bonds facilitates degradation, with an onset temperature of 177 °C (the earlier observed weight loss is associated with the evaporation of occluded water). The decomposition process occurs mainly via β-hydrogen elimination or homolytic cleavage of aliphatic bonds, leading to rapid mass loss. Derivative **P17**, containing a rigid single *p*-phenylene ring directly connected to sulfur atoms, exhibits significantly higher thermal stability than polymer **P16**. The onset decomposition temperature was determined to be 274 °C. Rigidification of the main chain through the introduction of an aromatic ring increases the activation energy of the degradation process. The decomposition process is clearly shifted toward higher temperatures due to the presence of more stable C_ar_-S bonds (resulting from the conjugation effect between the *π* electrons of the ring and the lone electron pairs on the sulfur atom). Derivative **P18**, containing an extended aromatic system composed of two phenylene rings connected by a sulfide bridge, is characterized by the highest onset decomposition temperature in the series (290 °C), and its decomposition process—especially at later stages—proceeds markedly more slowly. The high density of aromatic rings promotes thermal stabilization of the radicals formed during pyrolysis. In the process of high-temperature decomposition, this material is able to undergo intramolecular cyclization and secondary crosslinking, which slows down the release of volatile degradation products and results in a high residual mass at elevated temperatures.

Samples **P16**–**P18** were also subjected to DSC analysis in order to determine additional thermal parameters (glass transition, melting point, and crystallization temperatures). The recorded heat flow curves as a function of temperature for the second heating and cooling runs are presented in Figures S39–S41 (See ESI). Unfortunately, no changes in heat flow were observed in the temperature range from −30 to 180 °C, indicating their amorphous character.

### 2.3. The Preparative Scale of Synthesis of Product P1

**Scheme 5:**
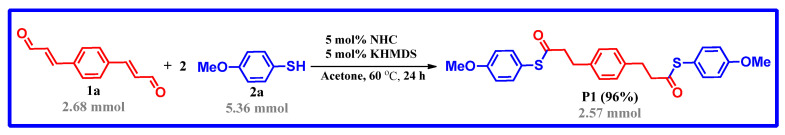
Scaled-up synthesis of **P1**.

Subsequently, to illustrate the synthetic utility of the developed protocol, a large-scale reaction of **1a** with **2a** was carried out (Scheme 5). To our great satisfaction, the reaction yielded the expected product **P1**, isolated in pure form in 96% yield. This result clearly demonstrates that the methodology possesses significant application potential.

### 2.4. Reaction Mechanism

**Scheme 6:**
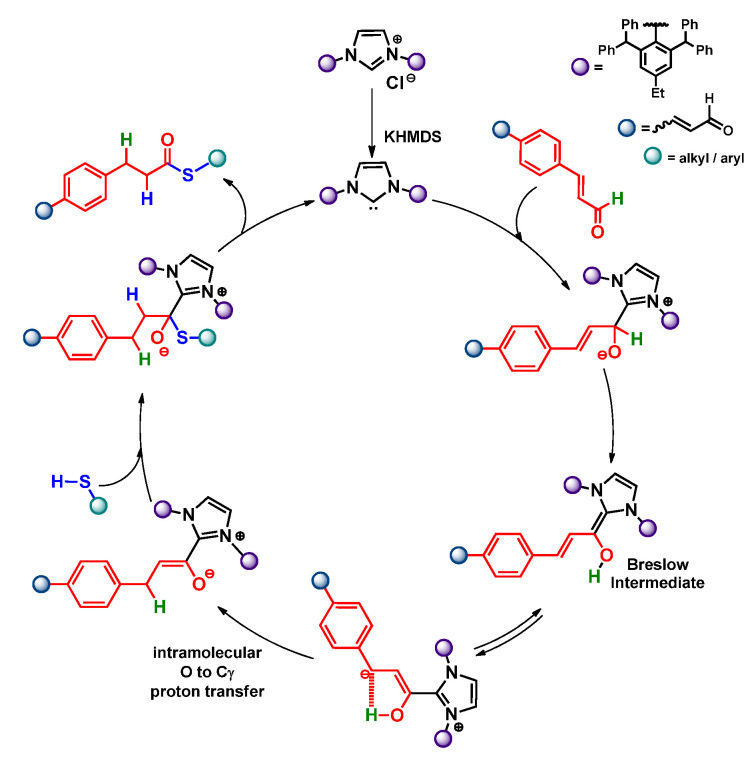
Proposed mechanism.

Based on our research on NHC-catalyzed thioesterification and the available literature, we propose the following mechanism for the described transformation [44,49,50,51,52,53,54,55] (Scheme 6). Deprotonation of the NHC precursor generates the free carbene, which adds to the α,β-unsaturated aldehyde to form the corresponding zwitterionic adduct. Following proton transfer, the Breslow intermediate is generated [56,57,58]. This species is in equilibrium with its homoenolate form [59]. Subsequent protonation at the β-position furnishes an enol intermediate that tautomerizes to the corresponding acyl azolium species [60,61]. Nucleophilic attack of the thiol on the acyl azolium intermediate then affords the thioester product with concomitant regeneration of the NHC catalyst.

## 3. Materials and Methods

### 3.1. General Methods and Chemicals

Unless otherwise indicated, all operations were carried out under argon. ^1^H NMR and ^13^C NMR spectra were recorded at 25 °C in CD Cl_3_ on a Varian 400 (Palo Alto, CA, USA) operating at 402.6 and 101.2 MHz, respectively. ^29^Si NMR were recorded on a Bruker Ascend 400 Nanobay (Bellerica, MA, USA) operating at 79.50 MHz. Chemical shifts are reported in ppm with reference to the residual solvent peaks for ^1^H and ^13^C NMR and to TMS for ^29^Si NMR. Thin layer chromatography (TLC) was conducted on plates coated with a 250 μm thick silica gel layer and column chromatography was performed on silica gel 60 (70–230 mesh). ESI-MS spectra were obtained using a Synapt Gs-S HDMS (Waters, Milford, MA, USA) mass spectrometer with electrospray ion source and quadrupole-time-of-flight analyzer with resolving power of FWMH 38,000. Acetonitrile was used as the sample solvent. The Capillary Voltage was set to 4.5 kV, the sampling was set to 40 and the source temperature was equal to 120 °C. The most abundant ions in the ESI-MS spectra were protonated or sodiated ions of desired products. Thermogravimetric analysis (TGA) of the prepared samples was carried out using a Q50-TGA thermobalance (TA Instruments, Inc., New Castle, DE, USA) under an N_2_ flow of 90 mL min^−1^. Samples (4–5 mg) loaded on a platinum pan were heated from RT to 800 °C at a rate of 10 °C min^−1^. Differential Scanning Calorimetry (DSC) of the prepared samples was carried out using a DSC-1 calorimeter (Mettler-Toledo International, Inc., Columbus, OH, USA) under an N_2_ flow of 30 mL min^−1^. Samples (3–7 mg) loaded to the 40 µL aluminum pan with a pierced lid were heated from −30 to 150 or 180 °C (depending on the sample) at a rate of 10 °C min^−1^.

NHC ligand was prepared according to the literature procedures [62]. All the other reagents were commercially available and used as received. The chemicals were purchased from the following sources: silsesquioxanes (Hybrid Plastic, Hattiesburg, MS, USA), thiols, (Chemat, Northridge, CA, USA), KHMDS (Sigma Aldrich, St. Louis, MO, USA), chloroform-d_1_ (Deutero, Kastellaun, Germany) and acetone, DCM, *n*-hexane (Fisher Chemical, Waltham, MA, USA). All the solvents used were dried over CaH_2_ prior to use and stored over 4Å molecular sieves under argon.

### 3.2. General Procedure for Synthesis of Products ***P1**–**P10***

An oven-dried 5 mL glass reactor equipped with a magnetic stirring bar was charged under argon with the NHC carbene precursor (13.10 mg, 1.34 × 10^−5^ mol), KHMDS (2.67 mg, 1.34 × 10^−5^ mol) and acetone (1 mL). The reaction mixture was stirred at 25 °C and after 30 min, **2a**–**j** (2 equiv., 5.36 × 10^−4^ mol) and **1a** (1 equiv., 50 mg, 2.68 × 10^−4^ mol) were added. The color change from navy blue to red to brown was observed within seconds. The mixture was stirred at 60 ^°^C for the next 15 min. After that, the solvent was evaporated under vacuum. The residue was purified by column chromatography on silica gel using dichloromethane or a 1:1 *v*/*v* mixture of *n*-hexane and dichloromethane as eluents. Evaporation of the solvents afforded analytically pure compounds.

### 3.3. General Procedure for the Synthesis of Products ***P11**–**P15***

An oven-dried 5 mL glass reactor equipped with a magnetic stirring bar was charged under argon with the NHC precursor (13.10 mg, 1.34 × 10^−5^ mol), KHMDS (2.67 mg, 1.34 × 10^−5^ mol) and acetone (1 mL). The reaction mixture was stirred at RT and after 30 min, two different thiols **2a**–**j** (1 equiv., 2.68 × 10^−4^ mol each) and **1a** (1 equiv., 50 mg, 2.68 × 10^−4^ mol) were added. The color change from navy blue to red to brown was observed within seconds. The mixture was stirred at 60 ^°^C for the next 15 min. After that, the solvent was evaporated under vacuum. The residue was purified by column chromatography on silica gel using gradient column chromatography with *v*/*v* mixture of *n*-hexane and dichloromethane in different *v*/*v* ratios (1:4, 1:2, 1:1, 2:1) as eluents. Evaporation of the solvents afforded analytically pure compounds.

### 3.4. General Procedure for the Synthesis of Products ***P16**–**P18***

An oven-dried 5 mL glass reactor equipped with a magnetic stirring bar was charged under argon with the NHC carbene precursor (13.10 mg, 1.34 × 10^−5^ mol), KHMDS (2.67 mg, 1.34 × 10^−5^ mol) and acetone (1 mL). The reaction mixture was stirred at RT and after 30 min, **3a**–**c** (1 equiv., 2.68 × 10^−4^ mol) and **1a** (1 equiv., 50 mg, 2.68 × 10^−4^ mol) were added. The color change from navy blue to red and then to brown was observed within seconds. The mixture was stirred at 60 ^°^C for the next 15 min. After that, the solvent was evaporated under vacuum. The residue was purified by precipitation using methanol. Evaporation of the solvents afforded analytically pure compounds.

### 3.5. Synthesis of Product P1 on a Preparative Scale

An oven-dried 5 mL glass reactor equipped with a magnetic stirring bar was charged under argon with the NHC precursor (130.10 mg, 1.34 × 10^−4^ mol), KHMDS (26.78 mg, 1.34 × 10^−4^ mol) and acetone (1 mL). The reaction mixture was stirred at 25 °C and after 30 min, **2a** (2 equiv., 0.66 mL, 5.36 × 10^−3^ mol) and **1a** (1 equiv., 500 mg, 2.68 × 10^−3^ mol) were added. The color change from navy blue to red to brown was observed within seconds. The mixture was stirred at 60 °C for the next 24 h. After that, the solvent was evaporated under vacuum. The residue was purified by column chromatography on silica gel using dichloromethane or a 1:1 *v*/*v* mixture of *n*-hexane and dichloromethane as eluents. Evaporation of the solvents afforded analytically pure compounds.

## 4. Conclusions

In summary, we have developed and optimized an efficient NHC-catalyzed protocol for the synthesis of functionalized acrolein-based derivatives from diacrylaldehyde and thiols. The catalytic system was successfully applied to various modifications of commercially available (2*E*,2′*E*)-3,3′-(1,4-phenylene)diacrylaldehyde, enabling the synthesis of both symmetric and asymmetric molecular derivatives, as well as functionalized silsesquioxanes and cross-linked polymers. The obtained polymeric materials were characterized as novel sulfur-containing networks exhibiting interesting thermal properties. The methodology also demonstrated excellent scalability, as evidenced by scaled-up reaction, and its utility was further confirmed through the formation of crosslinked polymeric materials. The main advantages of the proposed procedure include the absence of transition-metal complexes, the use of a green solvent (acetone), equimolar substrate ratios with complete conversion, short reaction time (15 min), a broad substrate scope, and facile isolation of the final products, thereby facilitating the elimination of additional operations and reduction in both production cost and time.

## Figures and Tables

**Table 1:** Optimization of the reaction conditions.

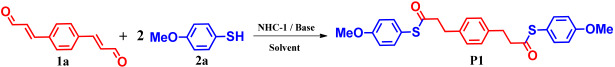						
Entry	Solvent	Temp. [°C]	NHC-1 [mol%]	Base	Time [h]	Conv. of 1a ^[d]^ [%]
1	Acetone	60	20	KHMDS	24	100
2	Acetone	60	15	KHMDS	24	100
3	Acetone	60	10	KHMDS	24	100
4	Acetone	60	5	KHMDS	24	100
5	Acetone	60	2.5	KHMDS	24	45
6	Acetone	60	-	KHMDS	72	0
7	Acetone	60	5	K_2_CO_3_	24	85
8	Acetone	60	5	*t*-BuOK	24	65
9	Acetone	60	5	NEt_3_	24	55
10	Acetone	60	5	-	24	2
11 ^[a]^	Acetone	60	5	KHMDS	24	100
12 ^[b]^	Acetone	60	5	KHMDS	24	100
13	*i*-PrOH	60	5	KHMDS	24	15
14	MiBK	60	5	KHMDS	24	35
15	EtOAc	60	5	KHMDS	24	28
16	Acetone	40	5	KHMDS	24	80
17	Acetone	25	5	KHMDS	24	65
18 ^[c]^	Acetone	60	5	KHMDS	24	5
19	Acetone	60	5	KHMDS	1.0	100
20	Acetone	60	5	KHMDS	0.5	100
21	**Acetone**	**60**	**5**	**KHMDS**	0.25	100
22	Acetone	60	5	KHMDS	0.125	58

Reaction conditions: Argon, [**1a**]:[**2a**] = 1:2, [**NHC-1**]:[Base] = 1:1; ^[a]^ Freshly isolated carbene was used as a catalyst; ^[b]^ [**NHC-1**]:[KHMDS] = 1:2; ^[c]^ Reaction conducted under air; ^[d]^ Determined by ^1^H NMR spectroscopy of the crude reaction mixture.

## Data Availability

All data generated or analyzed during this study are included in this published article (and its Supplementary Information files: Analytical data of isolated products, NMR spectra of isolated products: Figures S1–S31, XRD analysis: Table S1, Figure S32–S35, FT-IR spectra of isolated products: Figures S36–S38 and DSC spectra: Figures S39–S41) [63,64,65,66,67,68].

## Supplementary Materials

The following supporting information can be downloaded at: https://www.mdpi.com/article/10.3390/ijms27135941/s1.
