# Supplementary material for: Organocatalytic Thioesterification of a Conjugated α,β-Unsaturated Dialdehyde
Source: Int J Mol Sci. 2026 Jul 1;27(13):5941. doi: 10.3390/ijms27135941 (PMC13362346; doi:10.3390/ijms27135941)
Supplement: Supplementary file 1 [file ijms-27-05941-s001.zip › ijms-4362171-supplementary.pdf]

Supporting Information

for

Organocatalytic thioesterification of a conjugated α,β-unsaturated dialdehyde

Kamil Hanek,[a] Kacper Grzegorczyk,[a] Michał Dutkiewicz[b] and Patrycja Żak[a,*]

[a] *Adam Mickiewicz University in Poznan, Faculty of Chemistry, Uniwersytetu Poznańskiego 8, 61-614 Poznań, Poland. E-mail: pkw@amu.edu.pl*

[b] *Poznan Science and Technology Park, Adam Mickiewicz University Foundation, Rubiez 46, 61-612 Poznan, Poland.*

CONTENTS:

**1.**	**Analytical data of isolated compounds**	**S-2**
	1.1. Analytical data of symmetrical products	*S-2*
	1.2. Analytical data of unsymmetrical products	*S-3*
**2.**	**NMR spectra of isolated compounds**	**S-5**
	2.1. NMR spectra of symmetrical products	*S-5*
	2.2. NMR spectra of unsymmetrical products	*S-16*
**3.**	**XRD analysis**	**S-21**
**4.**	**FT-IR analysis**	**S-23**
**5.**	**DSC analysis**	**S-25**
**6.**	**Statistical analysis**	**S-26**
**7.**	**References**	**S-27**

1. Analytical data of isolated compounds

1.1. Analytical data of symmetrical products

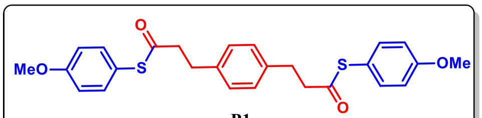 P1	Yellow solid, isolated yield: 93%; ^{1}H NMR (400 MHz, $CDCl_3$, 296K): δ (ppm): 2.91-2.95 (m, 4H, CH_2), 2.96-3.01 (m, 4H, CH_2), 3.82 (s, 6H, OCH_3), 6.93 (d, 4H, J_{HH} = 8.9 Hz, C_6H_4-OMe), 7.14 (s, 4H, C_6H_4), 7.29 (d, 4H, J_{HH} = 8.9 Hz, C_6H_4-OMe); ^{13}C NMR (100 MHz, $CDCl_3$, 296K): δ (ppm): 30.97 (CH_2), 44.83 (CH_2), 55.32 (OCH_3), 114.84, 118.33, 128.54, 136.05, 138.04, 160.61, 197.71 (CO); MS (ESI+): m/z 489 $[M+Na]^+$.
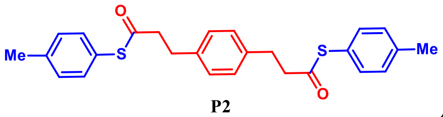 P2	White solid, isolated yield: 94%; ^{1}H NMR (400 MHz, $CDCl_3$, 296K): δ (ppm): 2.59 (s, 6H, CH_3), 3.09-3.13 (m, 4H, CH_2), 7.26 (s, 4H, C_6H_4), 7.32-7.35 (m, 4H, C_6H_4-Me), 7.38-7.40 (m, 4H, C_6H_4-Me); ^{13}C NMR (100 MHz, $CDCl_3$, 296K): δ (ppm): 21.31 (CH_3), 30.97 (CH_2), 45.00 (CH_2), 124.10, 128.53, 130.00, 134.00, 138.02, 139.65, 197.15 (CO); MS (ESI+): m/z 457 $[M+Na]^+$.
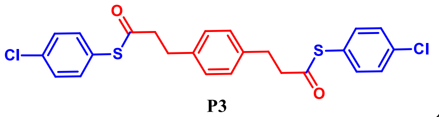 P3	Yellow solid, isolated yield: 91%; ^{1}H NMR (400 MHz, $CDCl_3$, 296K): δ (ppm): 2.91-3.03 (m, 8H, CH_2), 7.14 (s, 4H, C_6H_4), 7.28-7.32 (m, 4H, C_6H_4-Cl), 7.34-7.40 (m, 4H, C_6H_4-Cl); ^{13}C NMR (100 MHz, $CDCl_3$, 296K): δ (ppm): 30.88 (CH_2), 45.10 (CH_2), 125.98, 128.57, 129.42, 135.64, 135.80, 137.88, 196.10 (CO); MS (ESI+): m/z 497 $[M+Na]^+$.
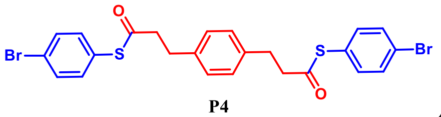 P4	Yellow solid, isolated yield: 95%; ^{1}H NMR (400 MHz, $CDCl_3$, 296K): δ (ppm): 2.91-3.01 (m, 8H, CH_2), 7.13 (s, 4H, C_6H_4), 7.22 (d, 4H, J_{HH} = 8.6 Hz, C_6H_4-Br), 7.52 (d, 4H, J_{HH} = 8.6 Hz, C_6H_4-Br); ^{13}C NMR (100 MHz, $CDCl_3$, 296K): δ (ppm): 30.89 (CH_2), 45.11 (CH_2), 124.04, 126.69, 128.56, 132.36, 135.84, 137.88, 195.85 (CO); MS (ESI+): m/z 482 $[M-Br]^+$.
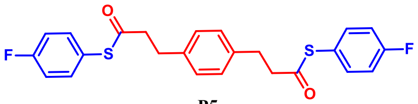 P5	White solid, isolated yield: 96%; ^{1}H NMR (400 MHz, $CDCl_3$, 296K): δ (ppm): 2.94-2.97 (m, 4H, CH_2), 2.98-3.01 (m, 4H, CH_2), 7.08-7.12 (m, 4H, C_6H_4-F), 7.15 (s, 4H, C_6H_4), 7.31-7.38 (m, 4H, C_6H_4-F); ^{13}C NMR (100 MHz, $CDCl_3$, 296K): δ (ppm): 30.90 (CH_2), 44.97 (CH_2), 116.44 (d, J = 22.0 Hz), 122.83, 128.55, 136.50 (d, J = 8.6 Hz), 137.93, 163.44 (d, J = 250.1 Hz), 196.59 (CO); MS (ESI+): m/z 465 $[M+Na]^+$.

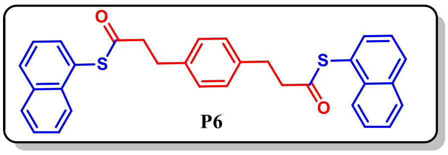 P6	White solid, isolated yield: 91%; ^{1}H NMR (400 MHz, $CDCl_3$, 296K): δ (ppm): 2.98-3.06 (m, 8H, CH_2), 7.18 (s, 4H, C_6H_4), 7.42 (dd, 2H, J_{HH} = 8.5, 1.8 Hz, CH_{Ar}), 7.50-7.55 (m, 4H, CH_{Ar}), 7.81-7.87 (m, 6H, CH_{Ar}), 7.90-7.94 (m, 2H, CH_{Ar}); ^{13}C NMR (100 MHz, $CDCl_3$, 296K): δ (ppm): 30.99 (CH_2), 45.17 (CH_2), 124.95, 126.53, 127.11, 127.76, 127.94, 128.60, 128.77, 130.87, 133.31, 133.51, 134.30, 138.02, 196.87 (*C*O); MS (ESI+): m/z 529 $[M+Na]^+$.
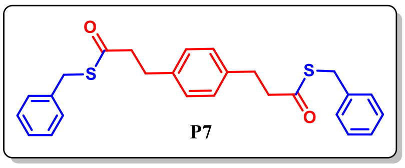 P7	Yellow solid, isolated yield: 94%; ^{1}H NMR (400 MHz, $CDCl_3$, 296K): δ (ppm): 2.82-2.88 (m, 4H, CH_2), 2.90-2.99 (m, 4H, CH_2), 4.12 (s, 4H, SCH_2), 7.08 (s, 4H, C_6H_4), 7.22-7.25 (m, 2H, C_6H_5), 7.27-7.32 (m, 8H, C_6H_5); ^{13}C NMR (100 MHz, $CDCl_3$, 296K): δ (ppm): 30.99 (CH_2), 33.18 (SCH_2), 45.22 (CH_2), 127.23, 128.40, 128.46, 128.58, 128.59, 128.79, 129.51, 137.54, 137.96, 197.82 (*C*O); MS (ESI+): m/z 457 $[M+Na]^+$.
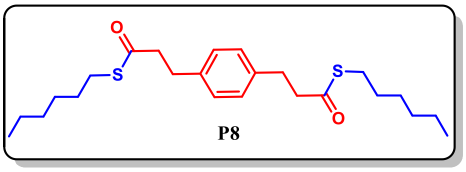 P8	Colorless oil, isolated yield: 95%; ^{1}H NMR (400 MHz, $CDCl_3$, 296K): δ (ppm): 0.88 (t, 6H, J_{HH} = 6.9 Hz, CH_3), 1.19-1.41 (m, 12H, CH_2), 1.44-1.66 (m, 6H, CH_2), 2.80-2.96 (m, 10H, CH_2), 7.10 (s, 4H, C_6H_4); ^{13}C NMR (100 MHz, $CDCl_3$, 296K): δ (ppm): 13.98 (CH_3), 22.48 (CH_2), 28.45 (CH_2), 28.91 (CH_2), 29.48 (CH_2), 31.08 (CH_2), 31.28 (CH_2), 45.52 (CH_2), 128.43, 129.52, 138.12, 198.72 (*C*O); MS (ESI+): m/z 445 $[M+Na]^+$.
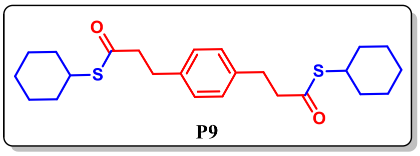 P9	White solid, isolated yield: 96%; ^{1}H NMR (400 MHz, $CDCl_3$, 296K): δ (ppm): 1.24-1.29 (m, 2H, CH_2), 1.34-1.45 (m, 8H, CH_2), 1.56-1.58 (m, 2H, CH_2), 1.65-1.72 (m, 4H, CH_2), 1.85-1.93 (m, 4H, CH_2), 2.75-2.84 (m, 4H, CH_2), 2.87-2.96 (m, 4H, CH_2), 3.47-3.54 (m, 2H, CH), 7.10 (s, 4H, C_6H_4); ^{13}C NMR (100 MHz, $CDCl_3$, 296K): δ (ppm): 25.33 (CH_2), 25.90 (CH_2), 31.08 (CH_2), 33.02 (CH_2), 42.26 (CH_2), 45.62 (CH_2), 51.77 (CH), 128.42, 129.33, 138.15, 198.44 (*C*O); MS (ESI+): m/z 441 $[M+Na]^+$.
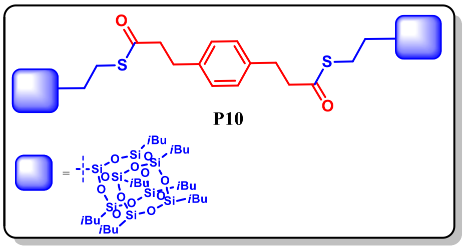 P10	White solid, isolated yield: 90%; ^{1}H NMR (400 MHz, $CDCl_3$, 296K): δ (ppm): 0.58-0.61 (m, 28H, CH_2), 0.66-0.69 (m, 4H, CH_2), 0.95 (d, 84H, J_{HH} = 4.2 Hz, CH_3), 1.64-1.68 (m, 2H, CH_2), 1.82-1.87 (m, 14H, CH), 2.80-2.83 (m, 4H, CH_2), 2.86-2.92 (m, 8H, CH_2), 7.10 (s, 4H, C_6H_4); ^{13}C NMR (100 MHz, $CDCl_3$, 296K): δ (ppm): 22.43 (CH_2), 22.53 (CH_2), 23.20 (CH_2), 23.84 (CH), 23.89 (CH), 25.69 (CH_3), 31.07 (CH_2), 31.66 (CH_2), 45.60 (CH_2), 126.32, 127.11, 128.42, 138.19, 198.30 (*C*O); ^{29}Si NMR (79 MHz, $CDCl_3$, 296K): δ (ppm): -67.63, -67.89, -68.17; MS (ESI+): m/z 1992 $[M+Na]^+$.

1.2. Analytical data of unsymmetrical products

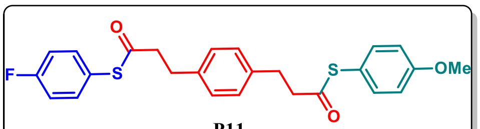 P11	White solid, isolated yield: 62%; ^{1}H NMR (400 MHz, $CDCl_3$, 296K): δ (ppm): 2.90-3.01 (m, 8H, CH_2), 3.82 (s, 3H, OCH_3), 6.93 (d, 4H, J_{HH} = 8.9 Hz, C_6H_4-OMe), 7.09-7.12 (m, 4H, C_6H_4-F), 7.14 (s, 4H, C_6H_4), 7.28 (d, 4H, J_{HH} = 8.9 Hz, C_6H_4-OMe), 7.32-7.37 (m, 4H, C_6H_4-F); ^{13}C NMR (100 MHz, $CDCl_3$, 296K): δ (ppm): 30.92 (CH_2), 30.97 (CH_2), 44.82 (CH_2), 45.01 (CH_2), 55.33 (OCH_3), 114.85, 116.45 (d, *J* = 22.1 Hz), 118.30, 128.08, 128.11, 128.53, 128.59, 129.36, 129.75, 136.06, 136.52 (d, *J* = 8.6 Hz), 137.84, 138.15, 160.63, 163.45 (d, *J* = 249.9 Hz), 196.66 (*C*O), 197.71 (*C*O); MS (ESI+): m/z 477 $[M+Na]^+$.
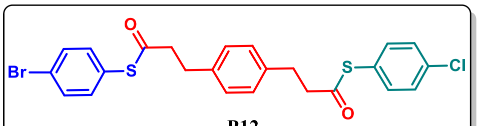 P12	White solid, isolated yield: 69%; ^{1}H NMR (400 MHz, $CDCl_3$, 296K): δ (ppm): 2.94-3.01 (m, 8H, CH_2), 7.14 (s, 4H, C_6H_4), 7.23 (d, 2H, J_{HH} = 8.6 Hz, C_6H_4-Br), 7.29 (d, 2H, J_{HH} = 8.7 Hz, C_6H_4-Cl), 7.37 (d, 2H, J_{HH} = 8.7 Hz, C_6H_4-Cl), 7.53 (d, 2H, J_{HH} = 8.6 Hz, C_6H_4-Br); ^{13}C NMR (100 MHz, $CDCl_3$, 296K): δ (ppm): 30.90 (CH_2), 45.11 (CH_2), 45.13 (CH_2), 124.07, 126.03, 126.66, 128.58, 129.41, 132.38, 135.64, 135.82, 135.86, 137.89, 137.90, 195.90 (*C*O), 196.06 (*C*O); MS (ESI+): m/z 543 $[M+Na]^+$.
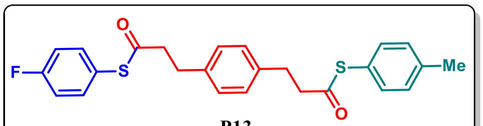 P13	White solid, isolated yield: 55%; ^{1}H NMR (400 MHz, $CDCl_3$, 296K): δ (ppm): 2.38 (s, 6H, CH_3), 2.92-3.02 (m, 8H, CH_2), 7.06-7.13 (m, 4H, C_6H_4-F), 7.13-7.17 (m, 4H, C_6H_4), 7.25-7.26 (m, 1H, C_6H_4-Me), 7.27-7.29 (m, 1H, C_6H_4-Me), 7.31-7.38 (m, 2H, C_6H_4-F); ^{13}C NMR (100 MHz, $CDCl_3$, 296K): δ (ppm): 21.32 (CH_3), 30.93 (CH_2), 30.98 (CH_2), 44.99 (CH_2), 45.02 (CH_2), 116.45 (d, *J* = 22.3 Hz), 122.90, 124.12, 128.55, 128.59, 130.02, 134.42, 136.52 (d, *J* = 8.5 Hz), 137.85, 138.04, 139.68, 163.45 (d, *J* = 250.1 Hz), 196.62 (*C*O), 197.16 (*C*O); MS (ESI+): m/z 461 $[M+Na]^+$.
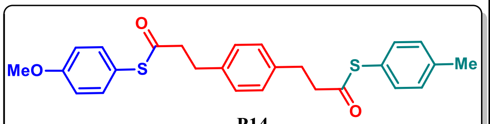 P14	White solid, isolated yield: 73%; ^{1}H NMR (400 MHz, $CDCl_3$, 296K): δ (ppm): 2.37 (s, 6H, CH_3), 2.90-3.01 (m, 8H, CH_2), 3.82 (s, 6H, OCH_3), 6.93 (d, 4H, J_{HH} = 8.9 Hz, C_6H_4-OMe), 7.14 (s, 4H, C_6H_4), 7.20-7.26 (m, 4H, C_6H_4-Me), 7.29 (d, 4H, J_{HH} = 8.9 Hz, C_6H_4-OMe); ^{13}C NMR (100 MHz, $CDCl_3$, 296K): δ (ppm): 21.32 (CH_3), 30.98 (CH_2), 44.84 (CH_2), 45.01 (CH_2), 55.33 (OCH_3), 114.85, 118.35, 124.11, 128.08, 128.11, 128.54, 130.01, 134.41, 136.06, 138.05, 139.63, 160.63, 197.15 (*C*O), 197.70 (*C*O); MS (ESI+): m/z 473 $[M+Na]^+$.

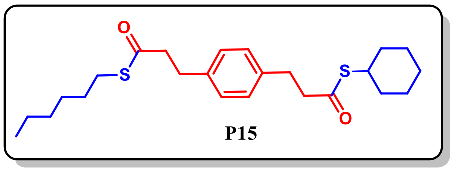	Colorless oil, isolated yield: 68%; ^{1}H NMR (400 MHz, $CDCl_3$, 296K): δ (ppm): 0.88 (t, 6H, J_{HH} = 6.9 Hz, CH_3), 1.12-1.50 (m, 12H, CH_2), 1.50-1.71 (m, 6H, CH_2), 1.74-1.98 (m, 2H, CH_2), 2.77-2.92 (m, 8H, CH_2), 5.29-5.32 (m, 1H, CH), 7.04-7.14 (m, 4H, C_6H_4); ^{13}C NMR (100 MHz, $CDCl_3$, 296K): δ (ppm): 14.15 (CH_3), 22.87 (CH_2), 25.89 (CH_2), 26.31 (CH_2), 28.80 (CH_2), 29.17 (CH_2), 31.31 (CH_2), 31.66 (CH_2), 33.38 (CH_2), 42.54 (CH_2), 45.76 (CH_2), 45.87 (CH), 128.50, 128.72, 129.64, 129.79, 129.99, 130.24, 138.60, 138.66, 198.45 (CO), 198.76 (CO); MS (ESI+): m/z 443 $[M+Na]^+$.

2. NMR spectra of compounds

2.1. NMR spectra of symmetrical products

Product P1

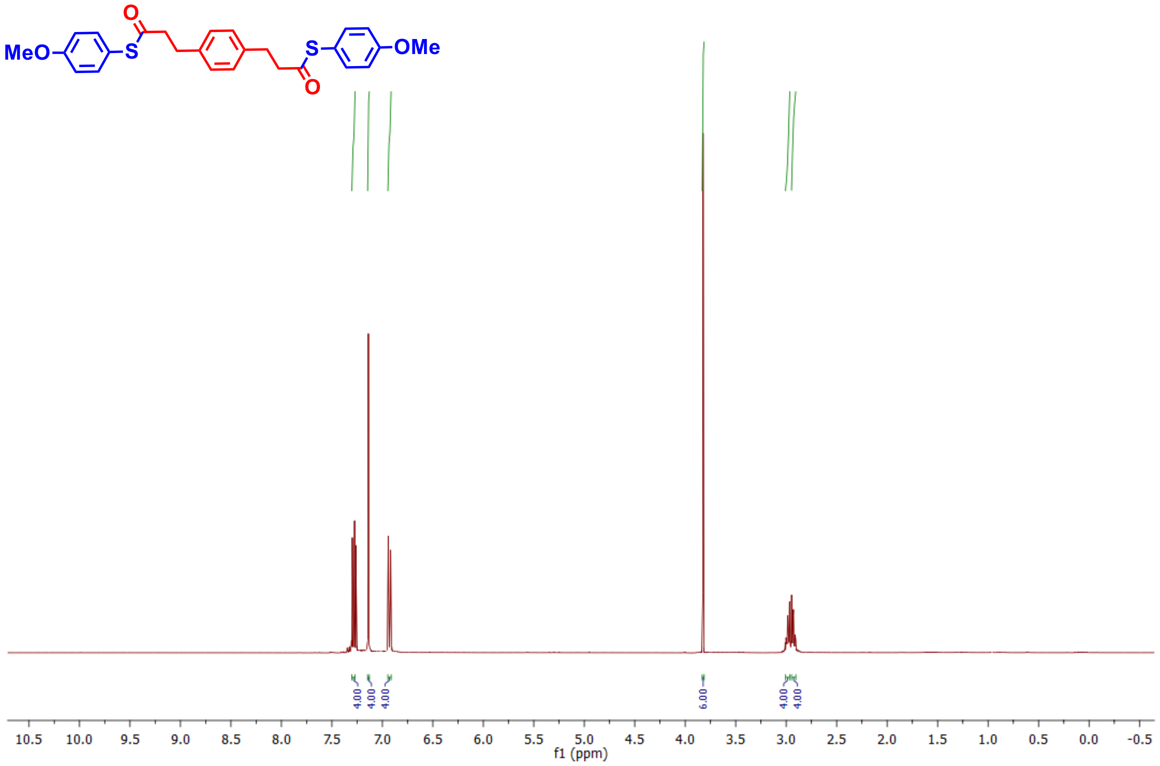

Figure S1. ^{1}H NMR (400 MHz, $CDCl_3$) of product **P1**

Product P1

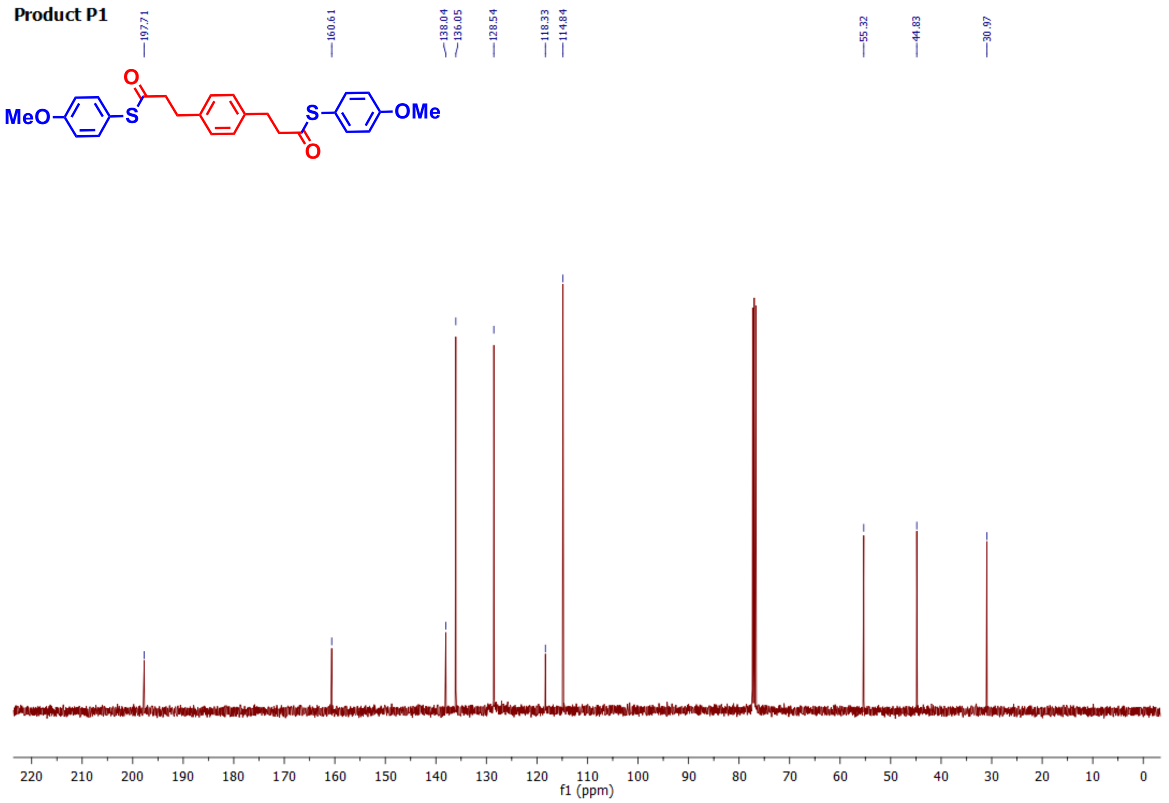

Figure S2. ^{13}C NMR (101 MHz, $CDCl_3$) of product **P1**

Product P2

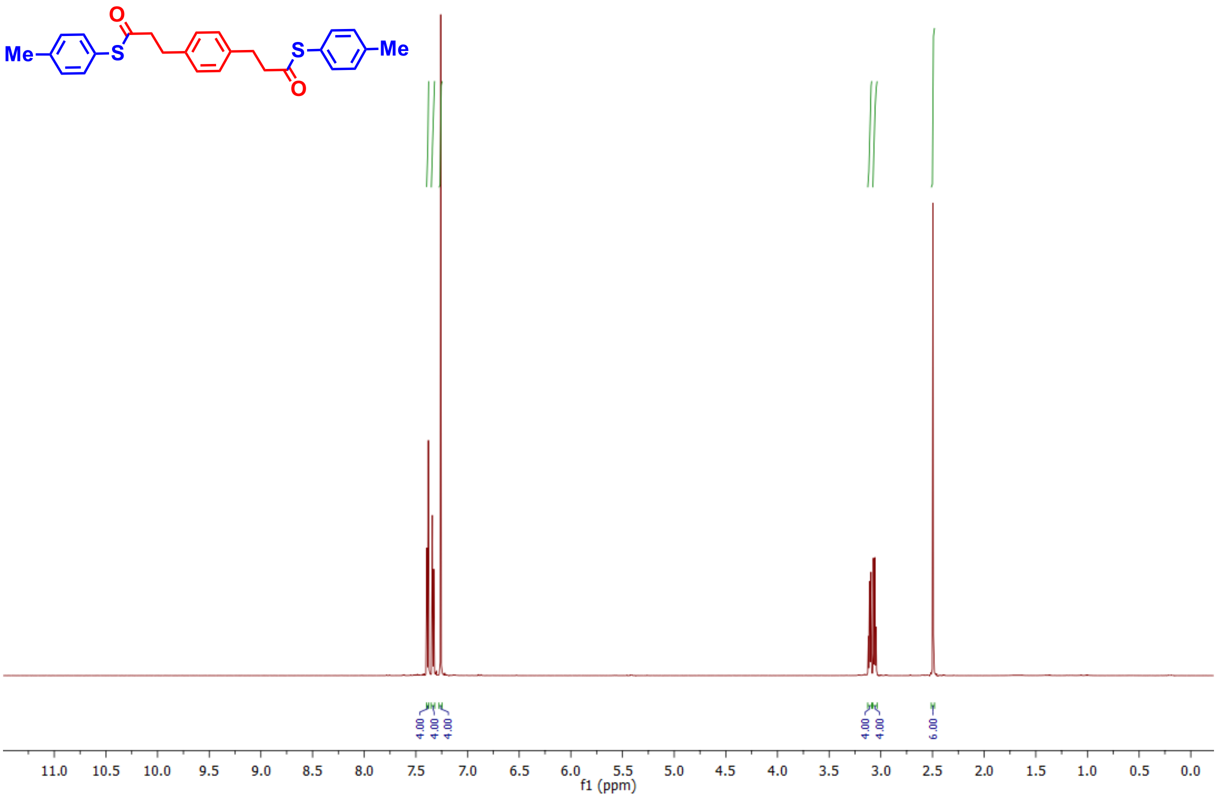

Figure S3. ^{1}H NMR (400 MHz, $CDCl_3$) of product **P2**

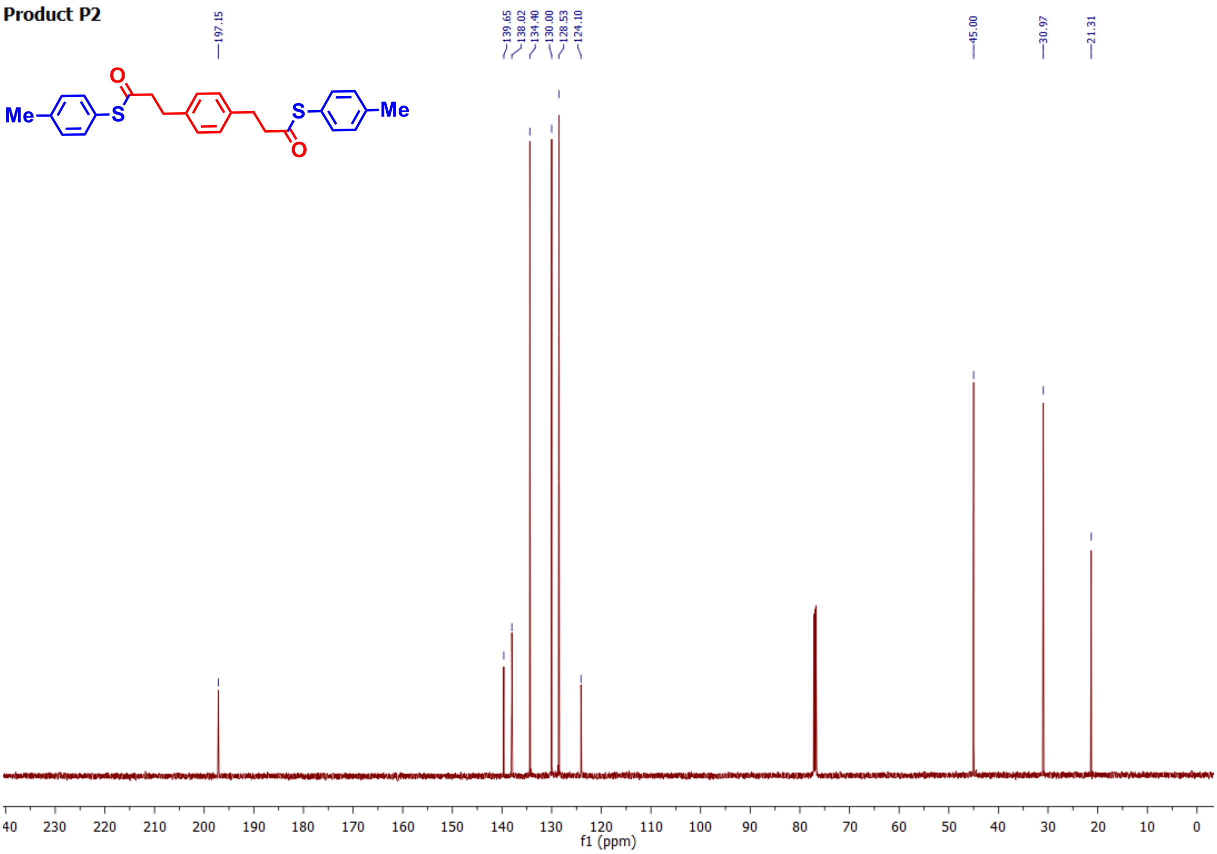

Figure S4. ^{13}C NMR (101 MHz, $CDCl_3$) of product **P2**

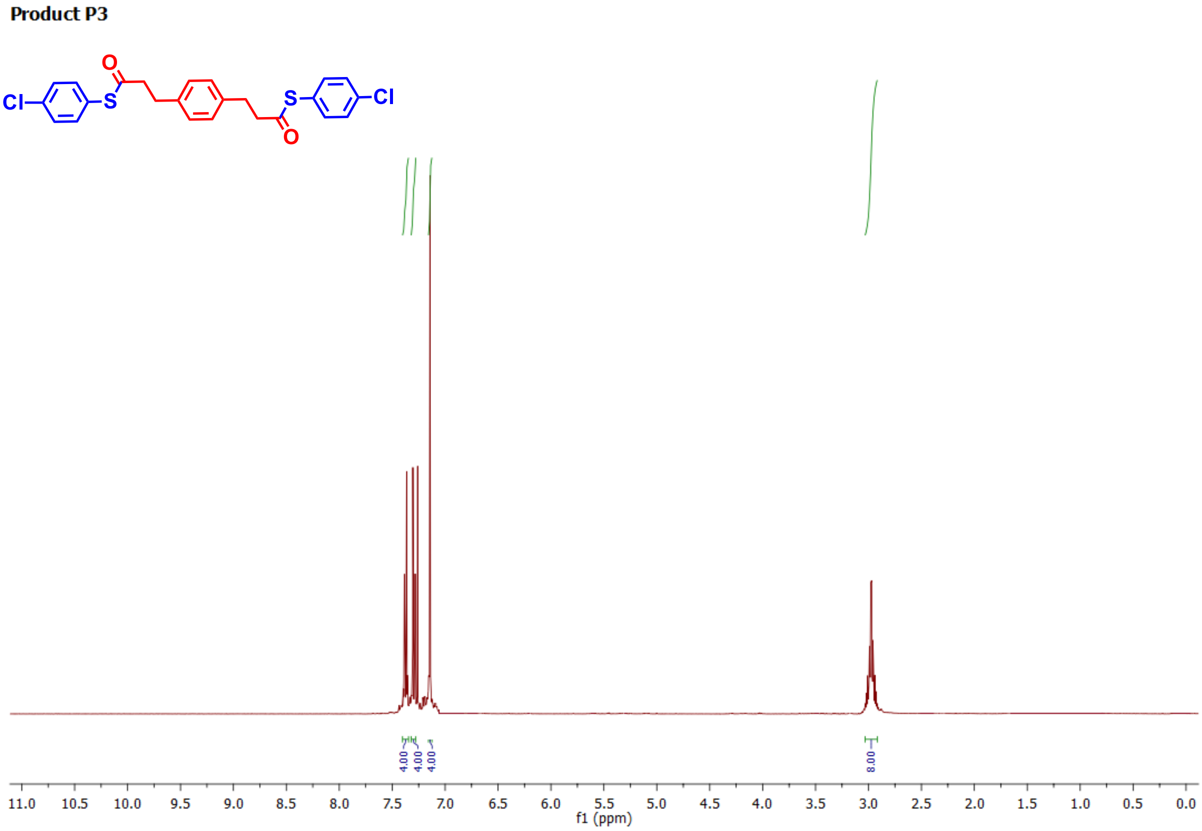

Figure S5. ^{1}H NMR (400 MHz, $CDCl_3$) of product **P3**

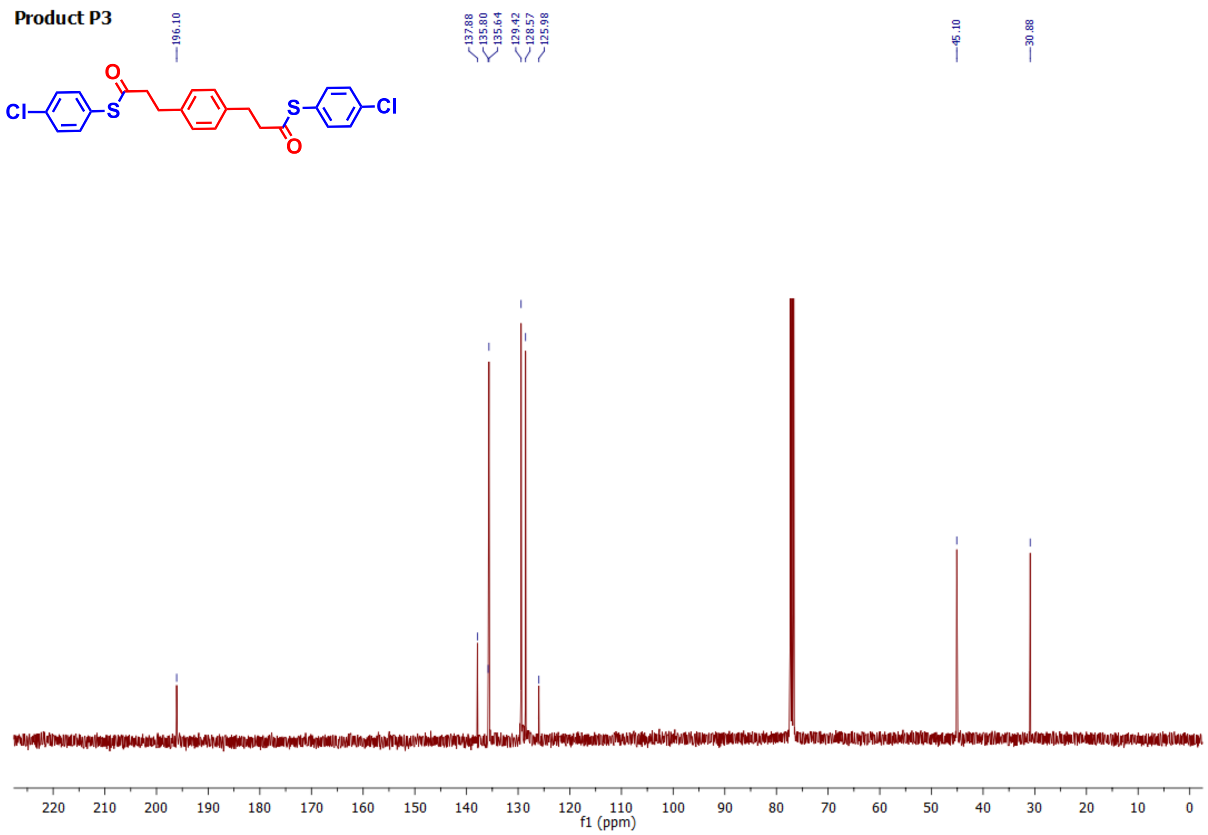

Figure S6. ^{13}C NMR (101 MHz, $CDCl_3$) of product **P3**

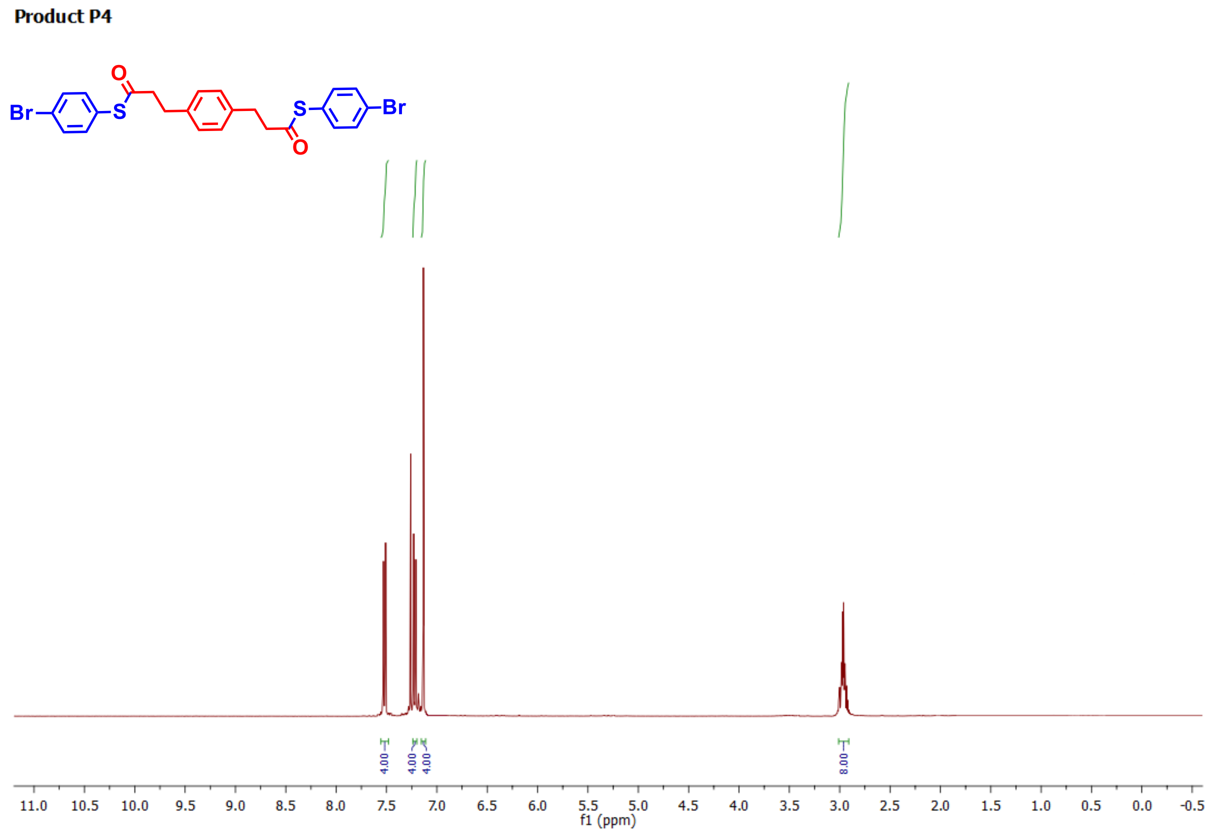

Figure S7. ^{1}H NMR (400 MHz, $CDCl_3$) of product **P4**

Product P4

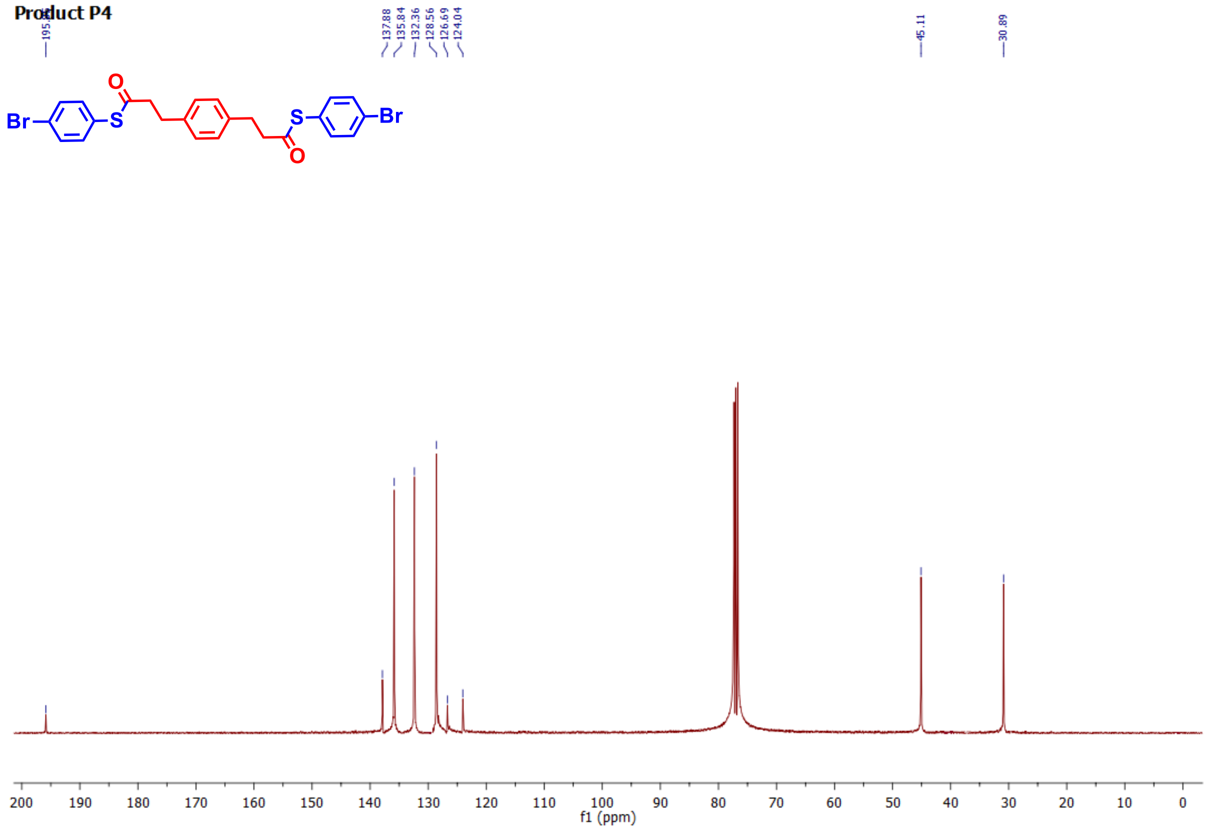

Figure S8. ^{13}C NMR (101 MHz, $CDCl_3$) of product **P4**

Product P5

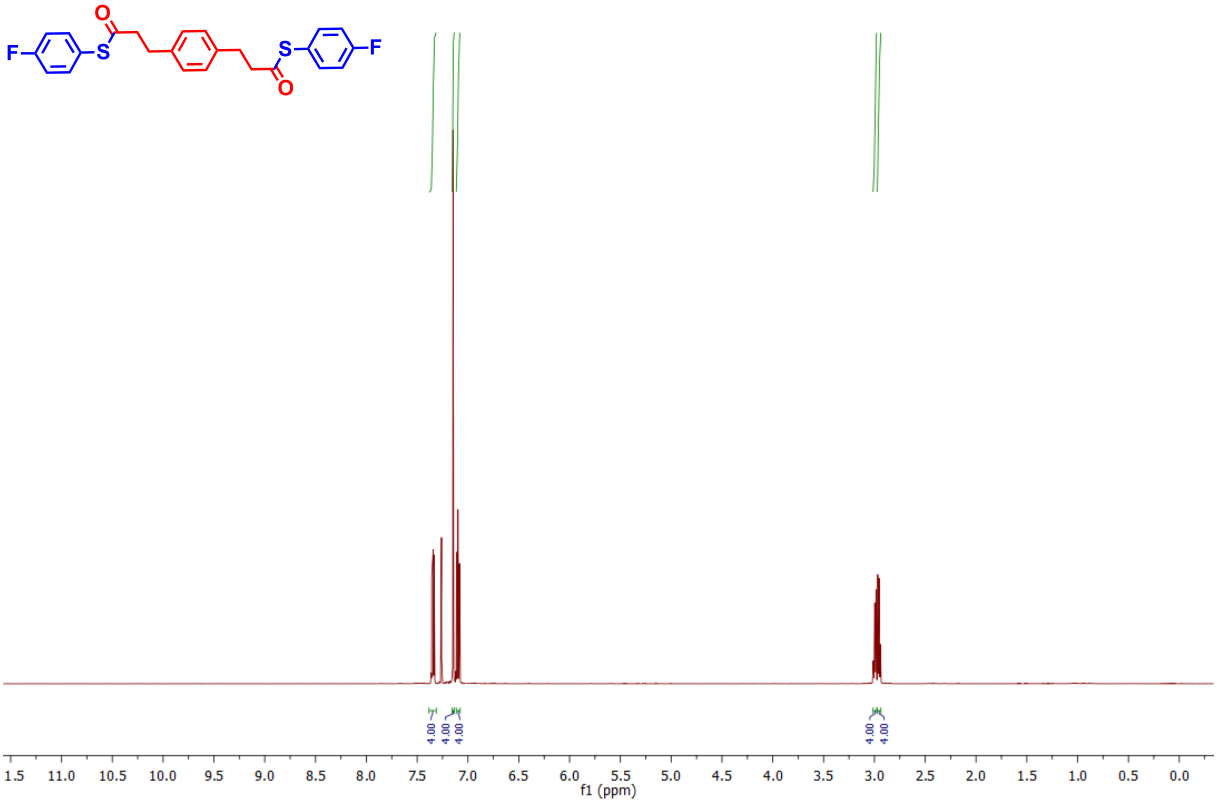

Figure S9. ^{1}H NMR (400 MHz, $CDCl_3$) of product **P5**

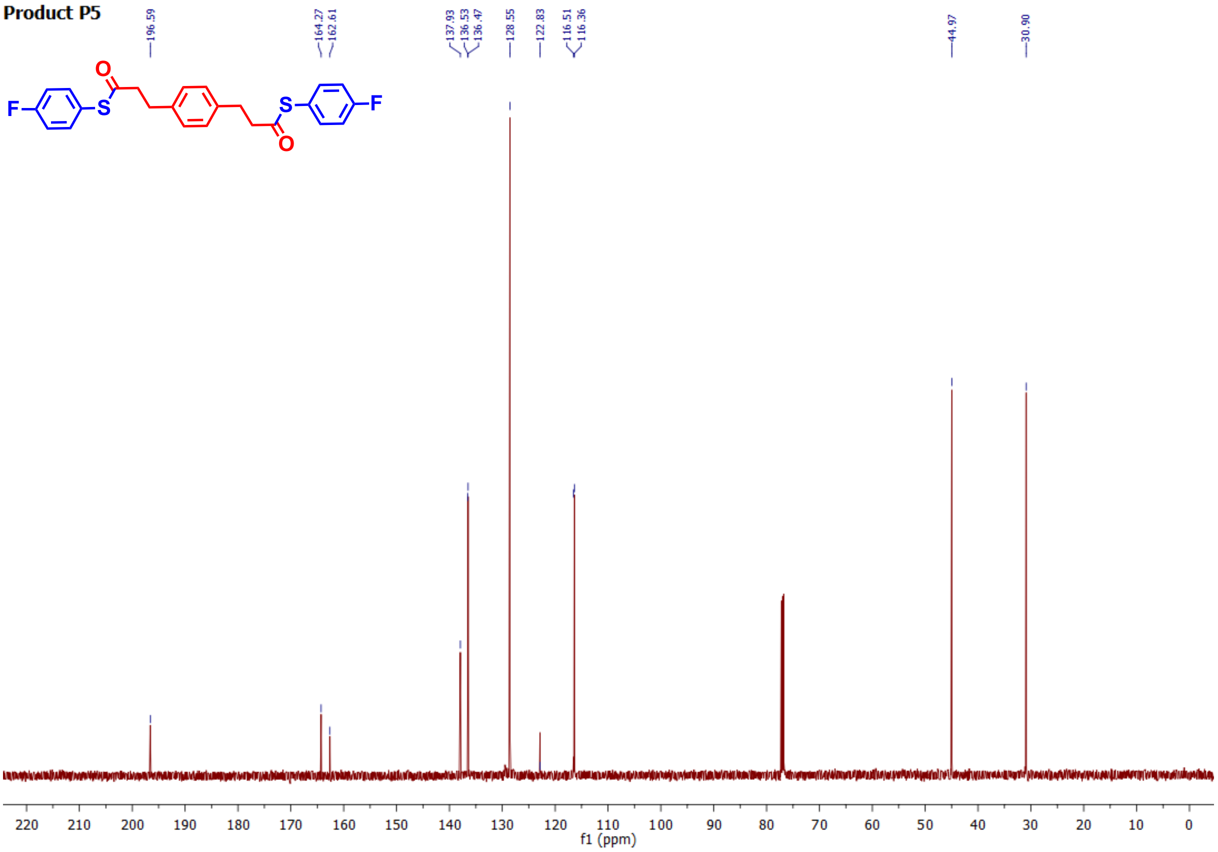

Figure S10. ^{13}C NMR (101 MHz, $CDCl_3$) of product **P5**

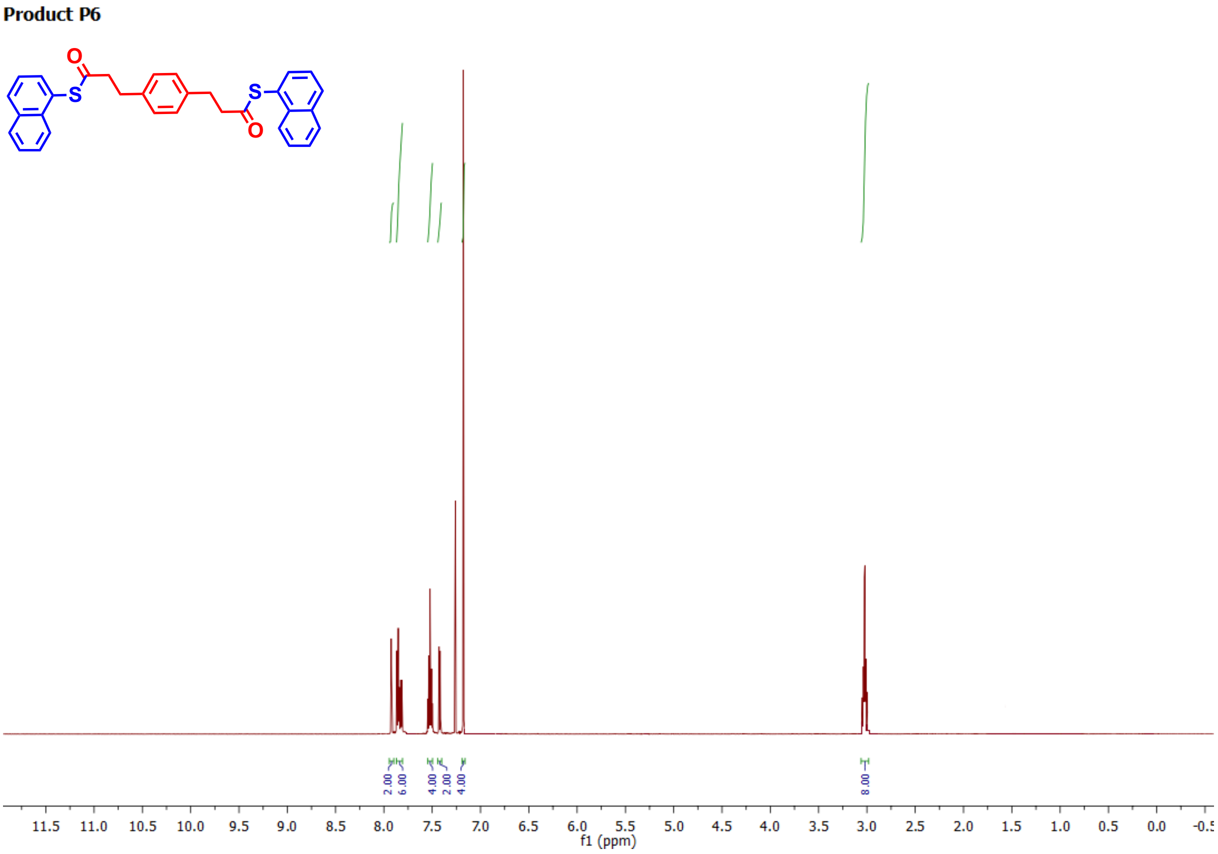

Figure S11. ^{1}H NMR (400 MHz, $CDCl_3$) of product **P6**

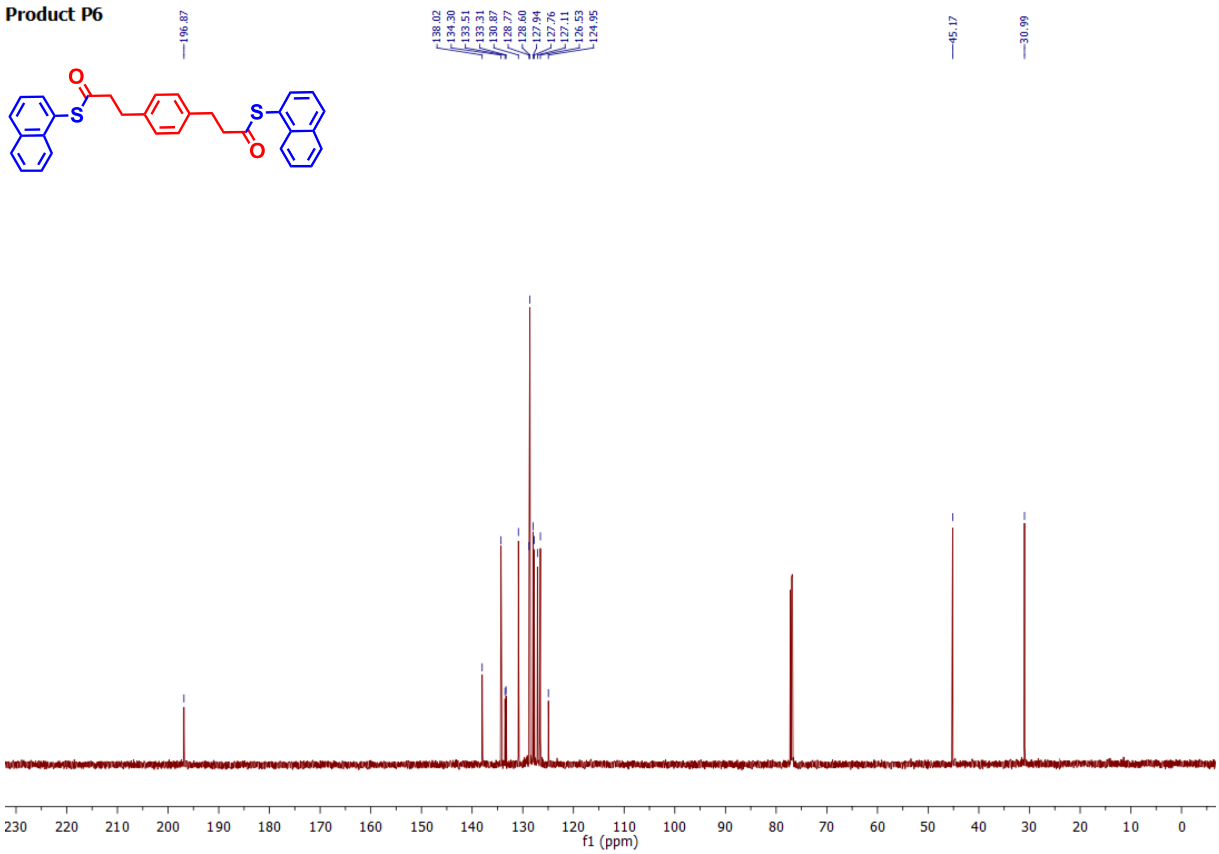

Figure S12. ^{1}H NMR (400 MHz, $CDCl_3$) of product **P6**

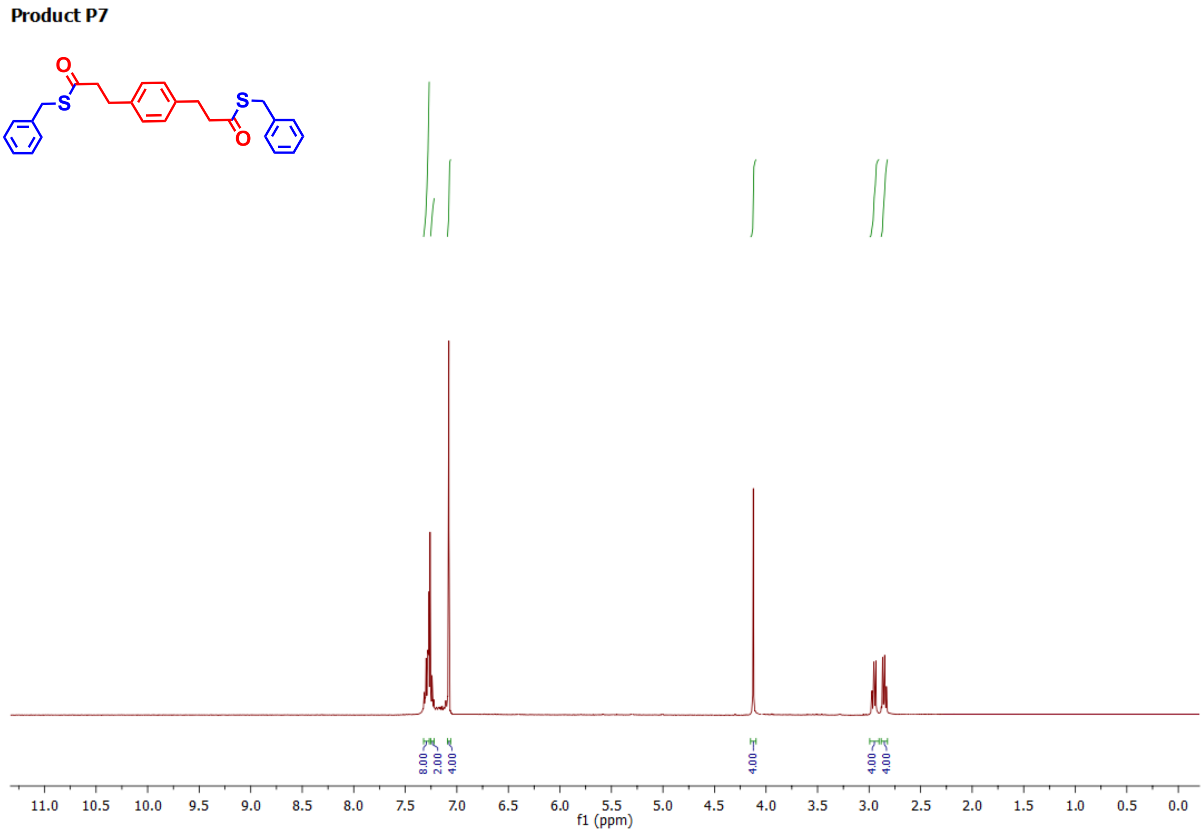

Figure S13. ^{1}H NMR (400 MHz, $CDCl_3$) of product **P7**

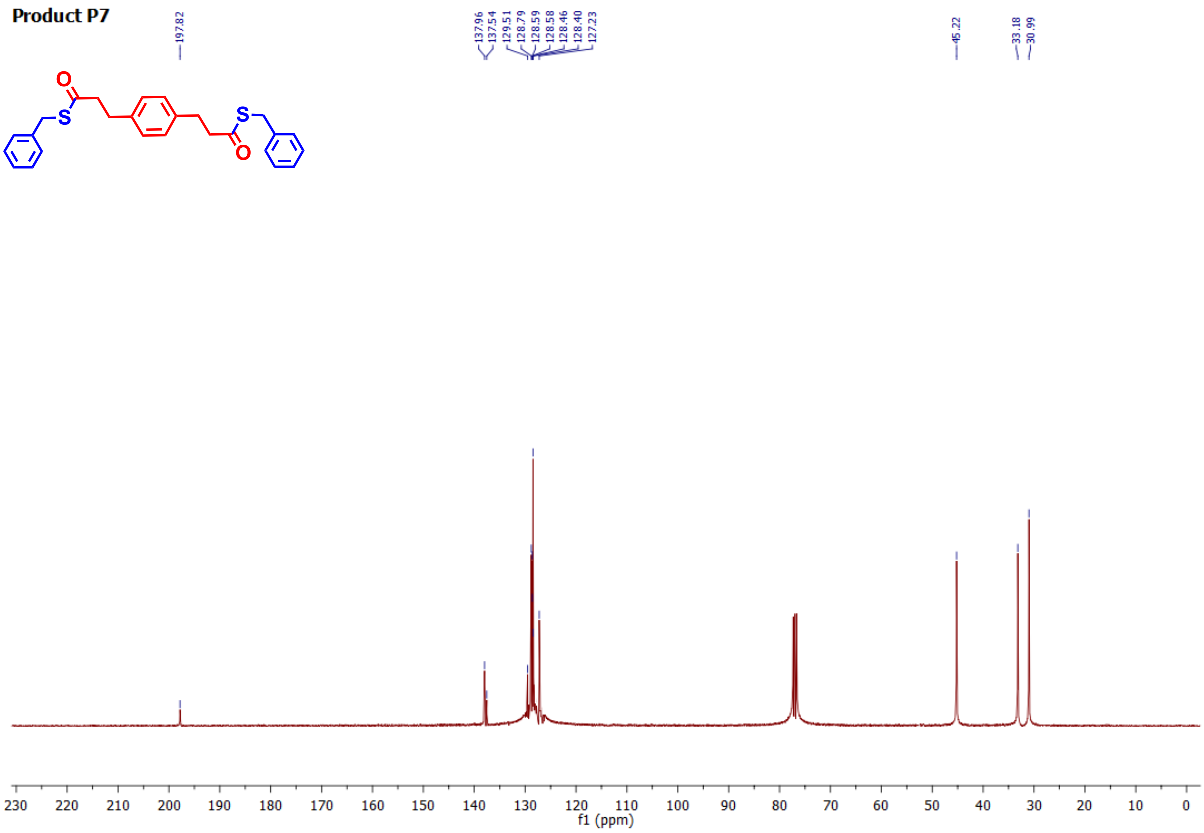

Figure S14. ^{13}C NMR (101 MHz, $CDCl_3$) of product **P7**

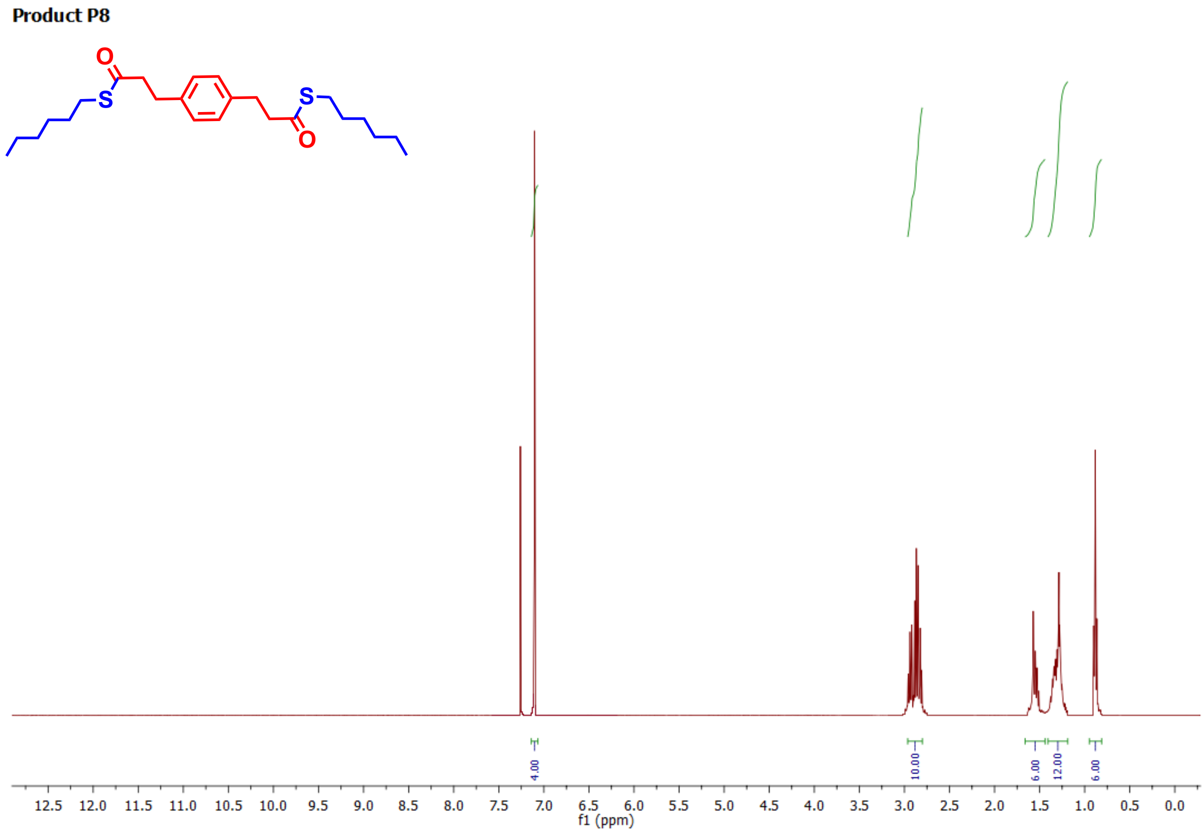

Figure S15. ^{1}H NMR (400 MHz, $CDCl_3$) of product **P8**

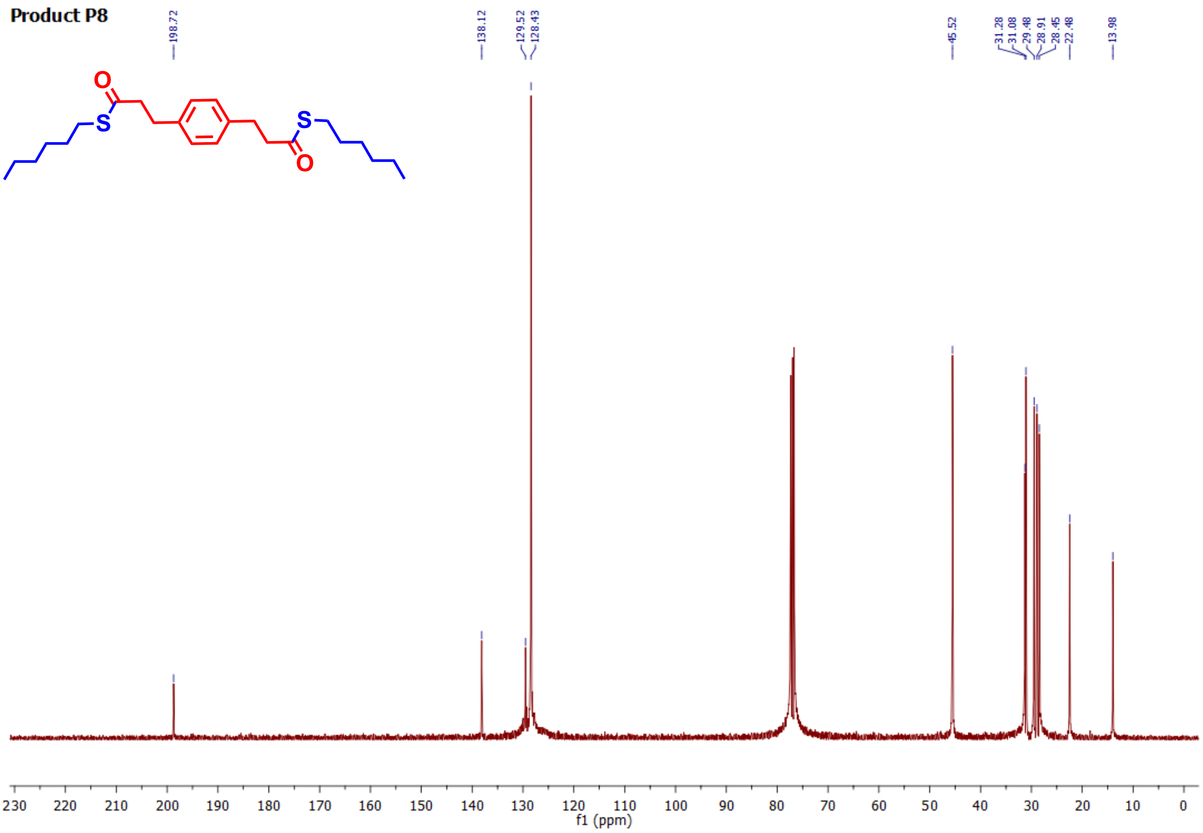

Figure S16. ^{13}C NMR (101 MHz, $CDCl_3$) of product **P8**

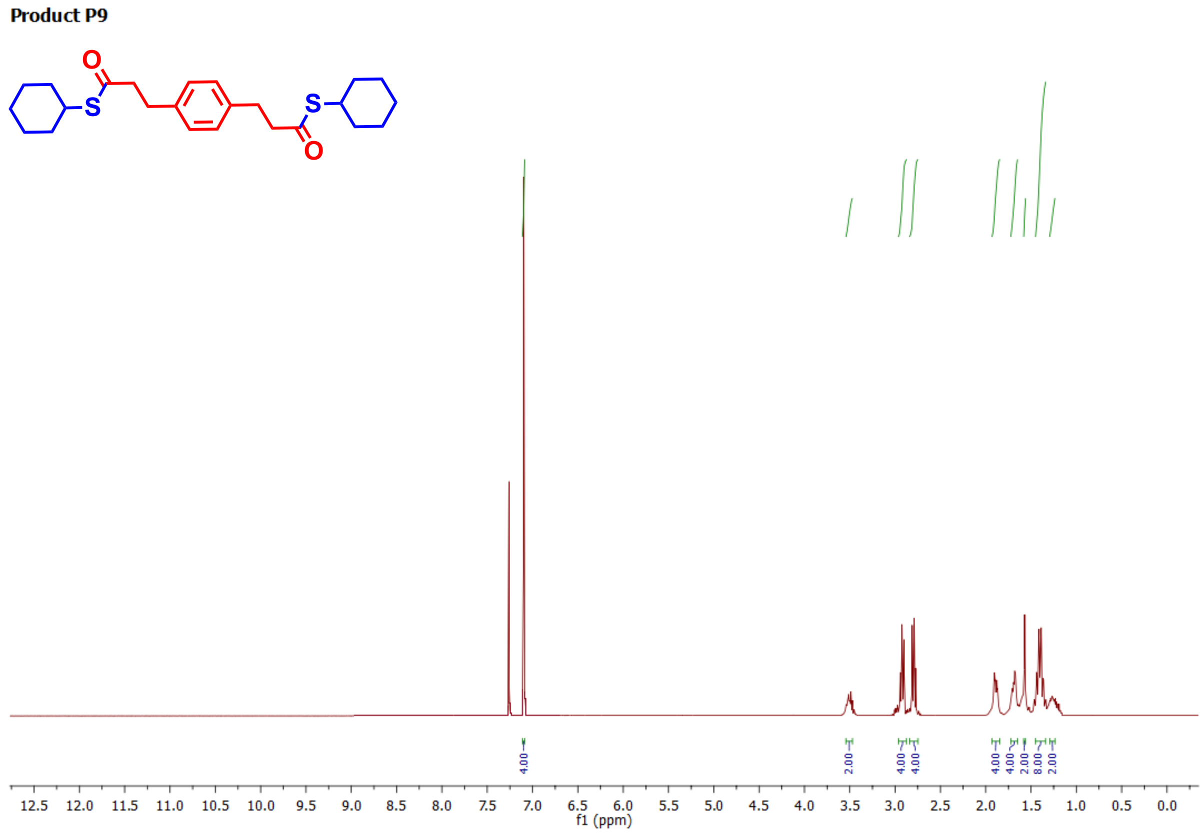

Figure S17. ^{1}H NMR (400 MHz, $CDCl_3$) of product **P9**

Product P9

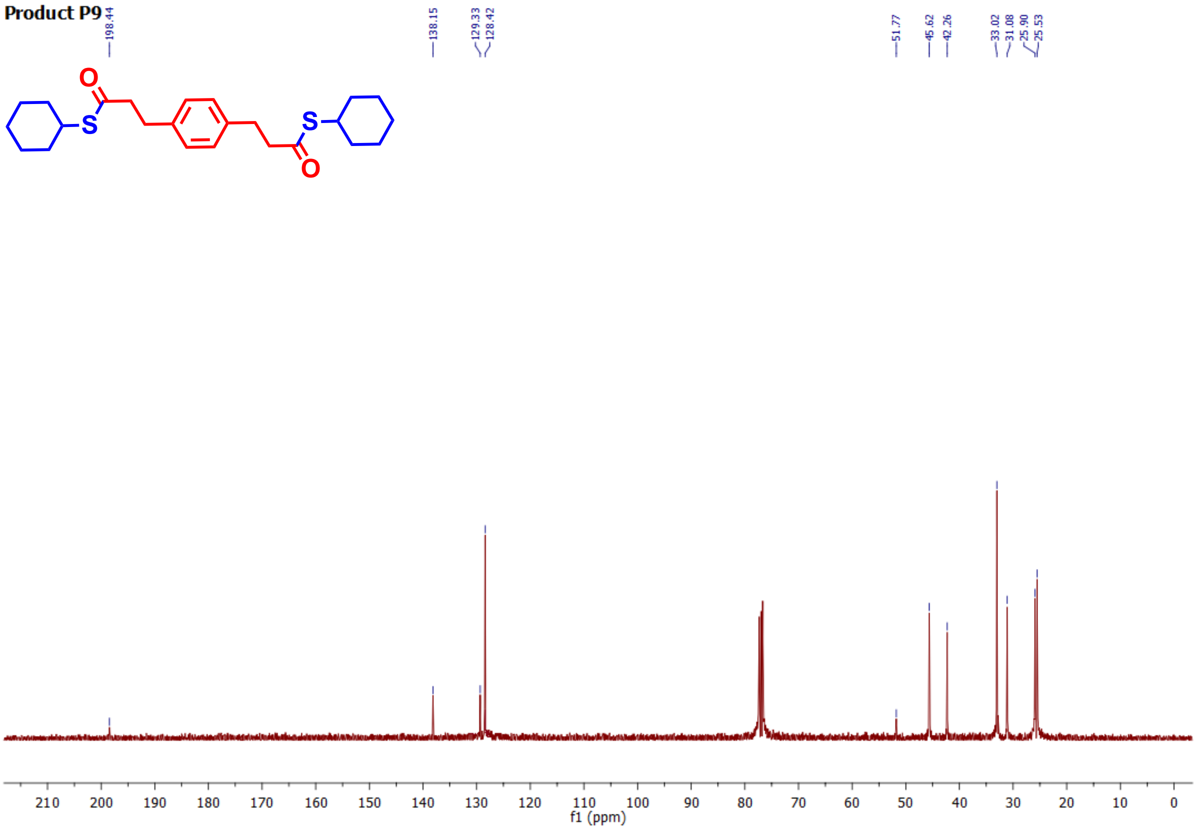

Figure S18. ^{13}C NMR (101 MHz, $CDCl_3$) of product **P9**

Product P10

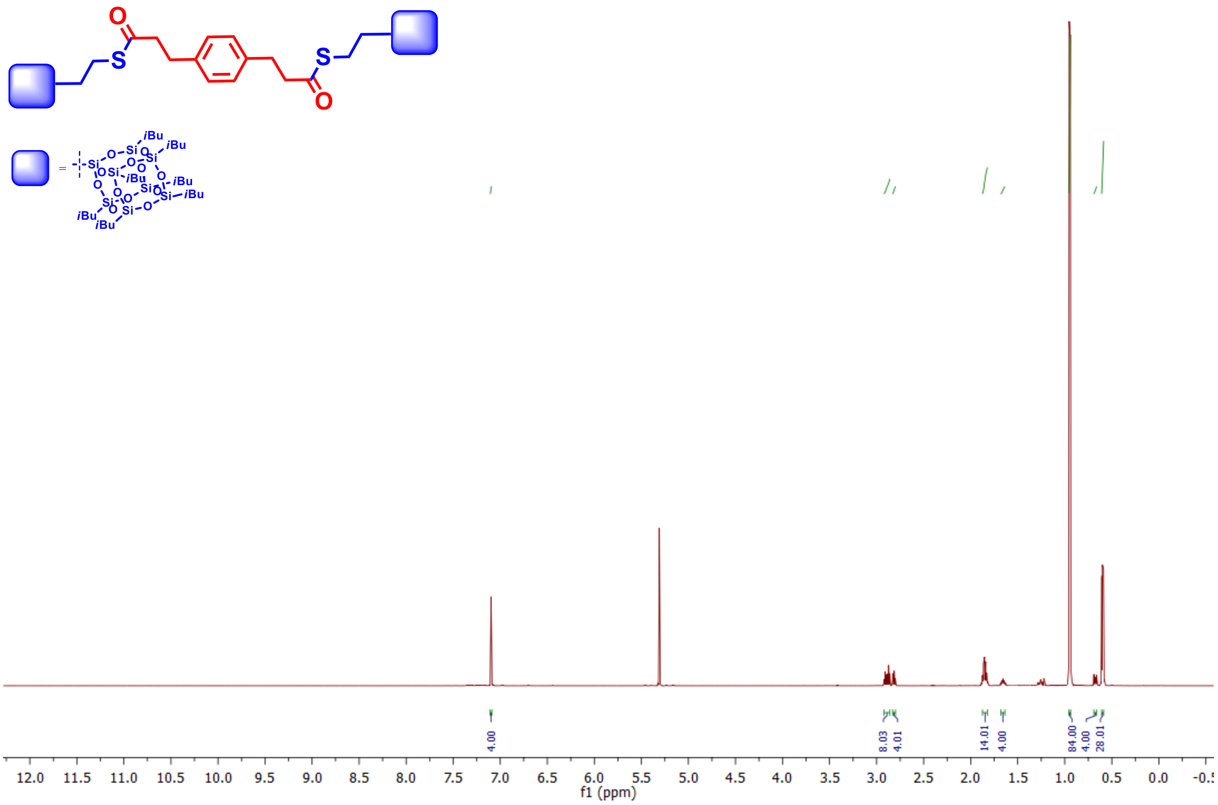

Figure S19. ^{1}H NMR (400 MHz, $CDCl_3$) of product **P10**

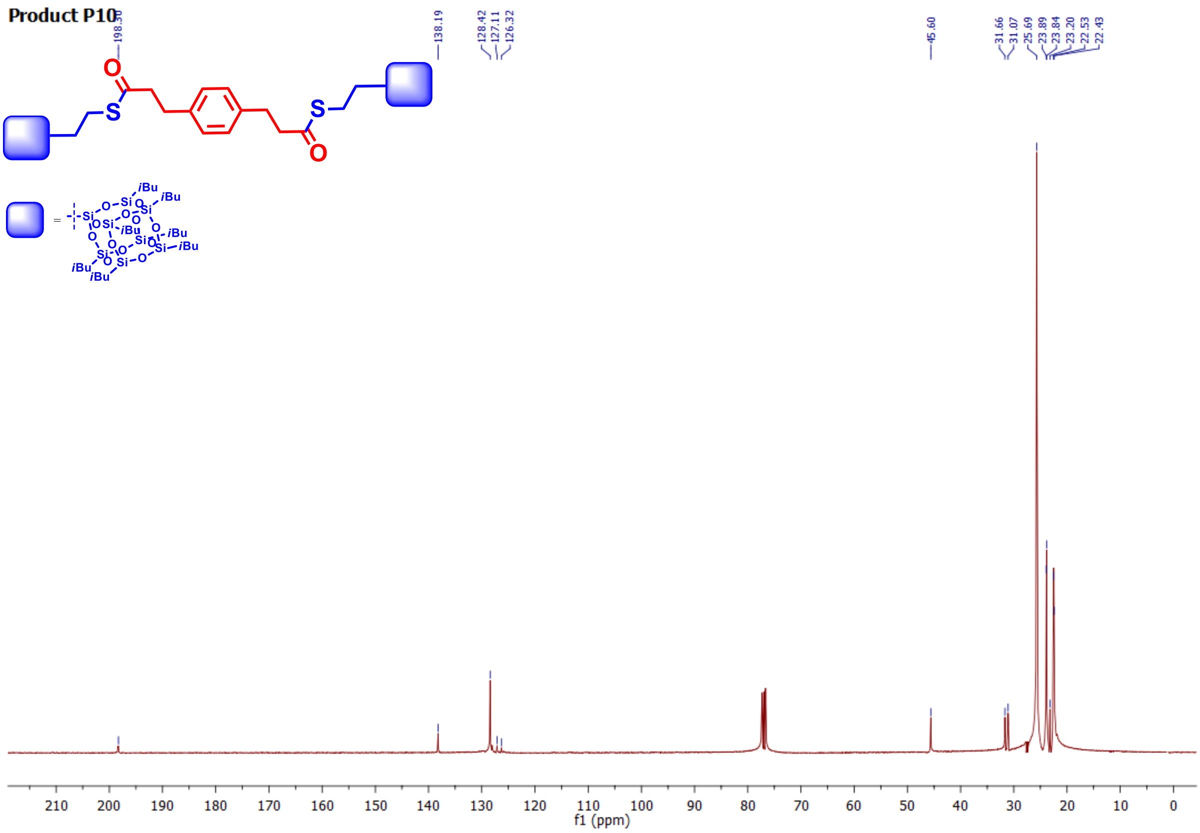

Figure S20. ^{13}C NMR (101 MHz, $CDCl_3$) of product **P10**

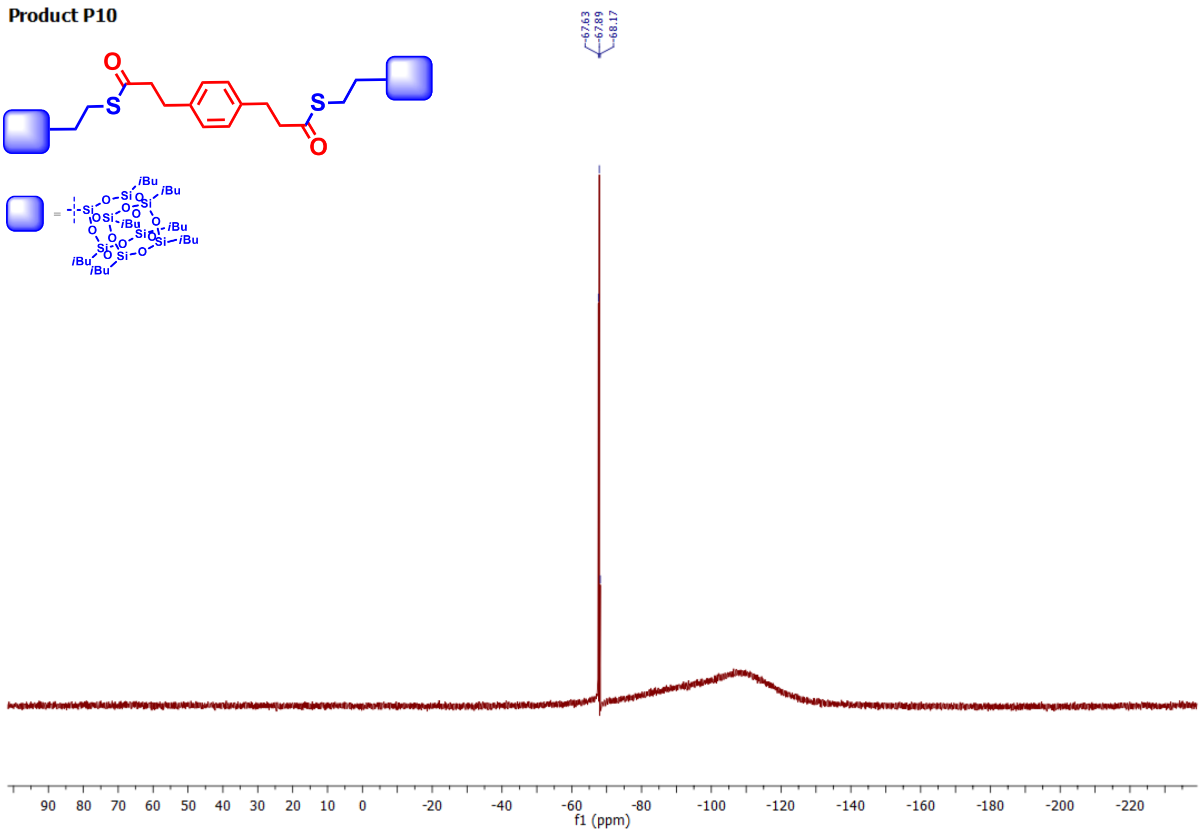

Figure S21. ^{29}Si NMR (79 MHz, $CDCl_3$) of product **P10**

2.2. NMR spectra of unsymmetrical products

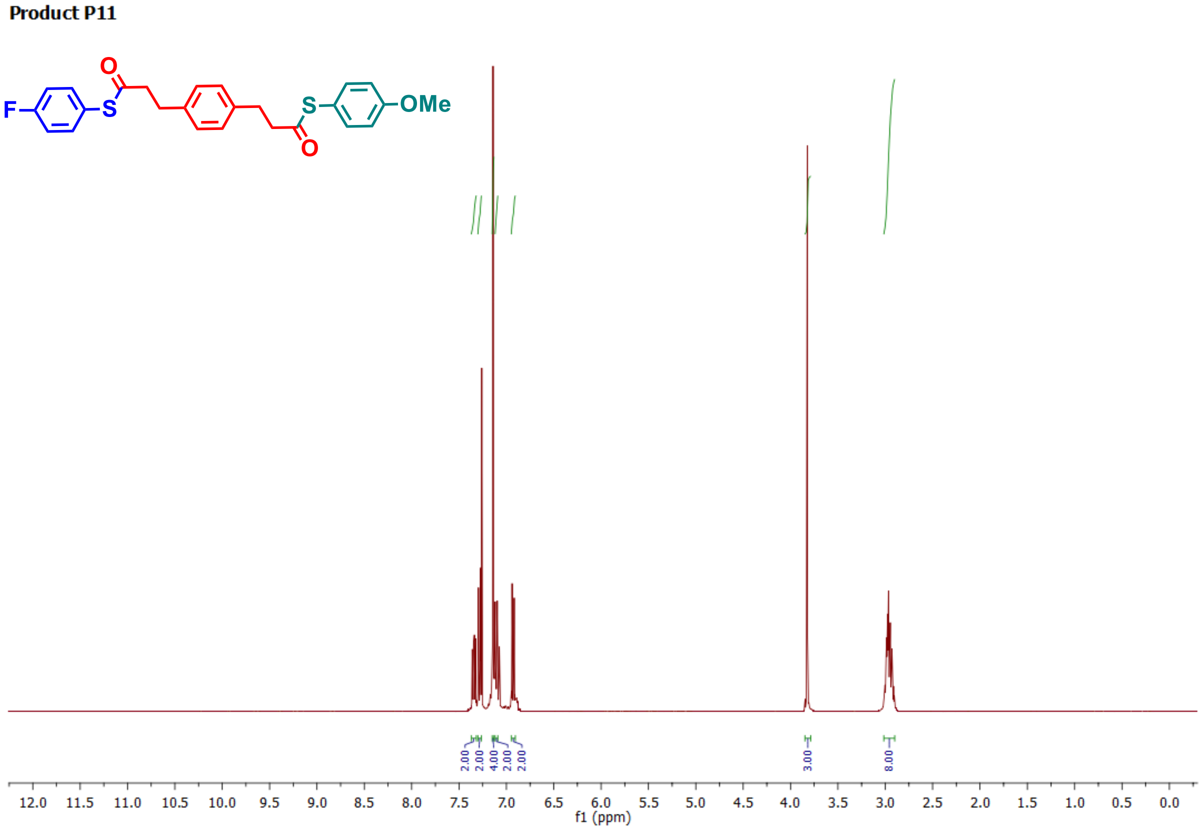

Figure S22. ^{1}H NMR (400 MHz, $CDCl_3$) of product **P11**

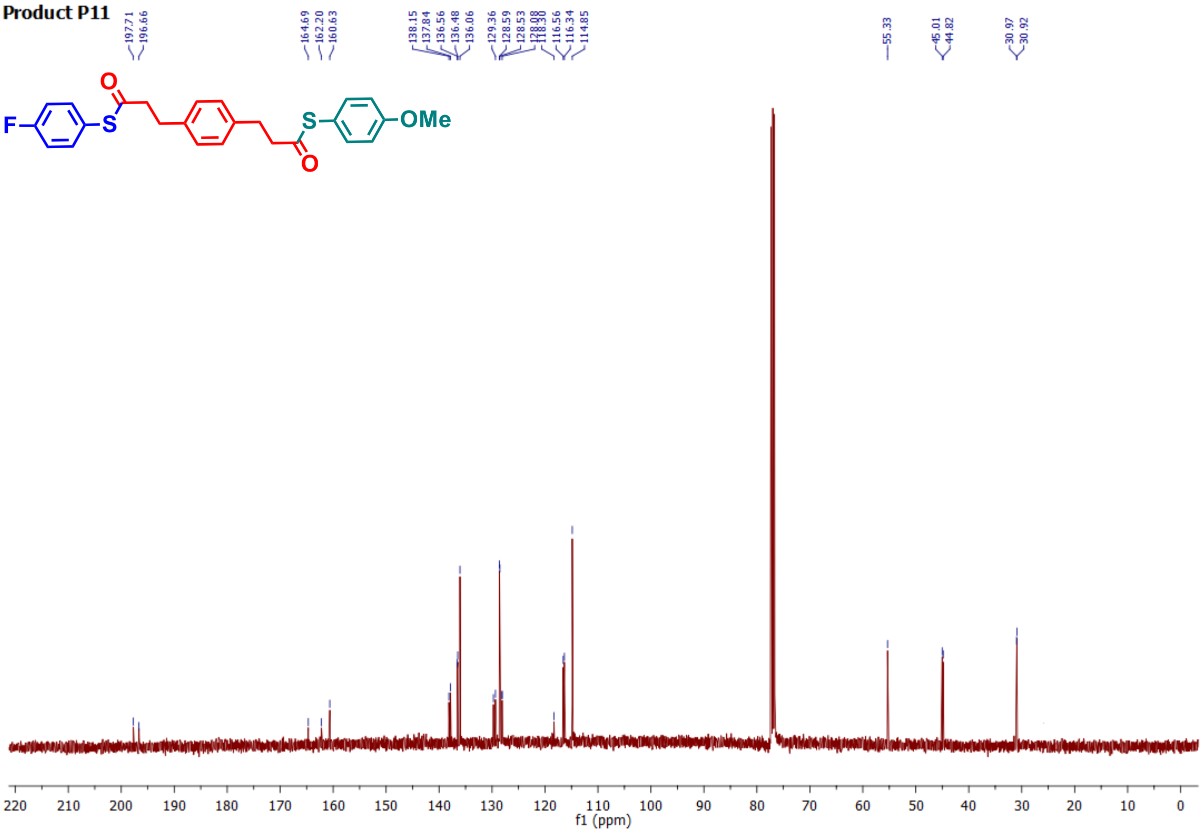

Figure S23. ^{13}C NMR (101 MHz, $CDCl_3$) of product **P11**

Product P12

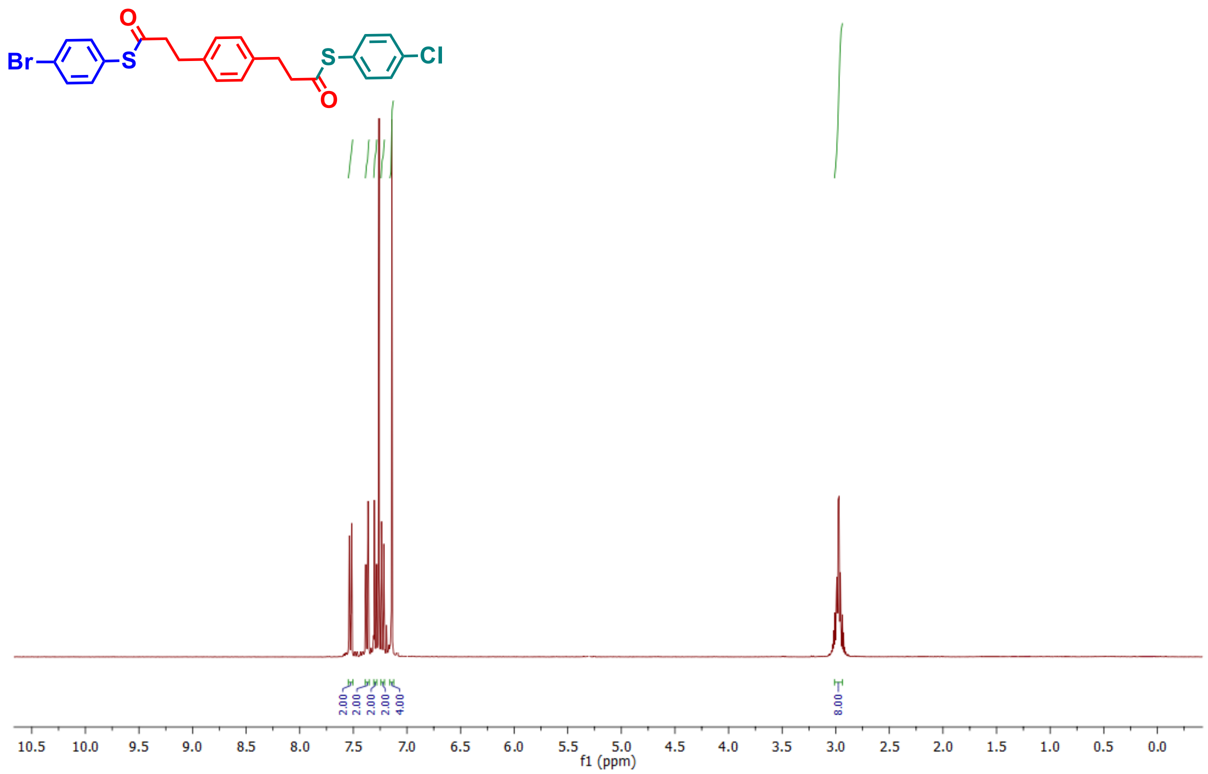

Figure S24. ^{1}H NMR (400 MHz, $CDCl_3$) of product **P12**

Product P12

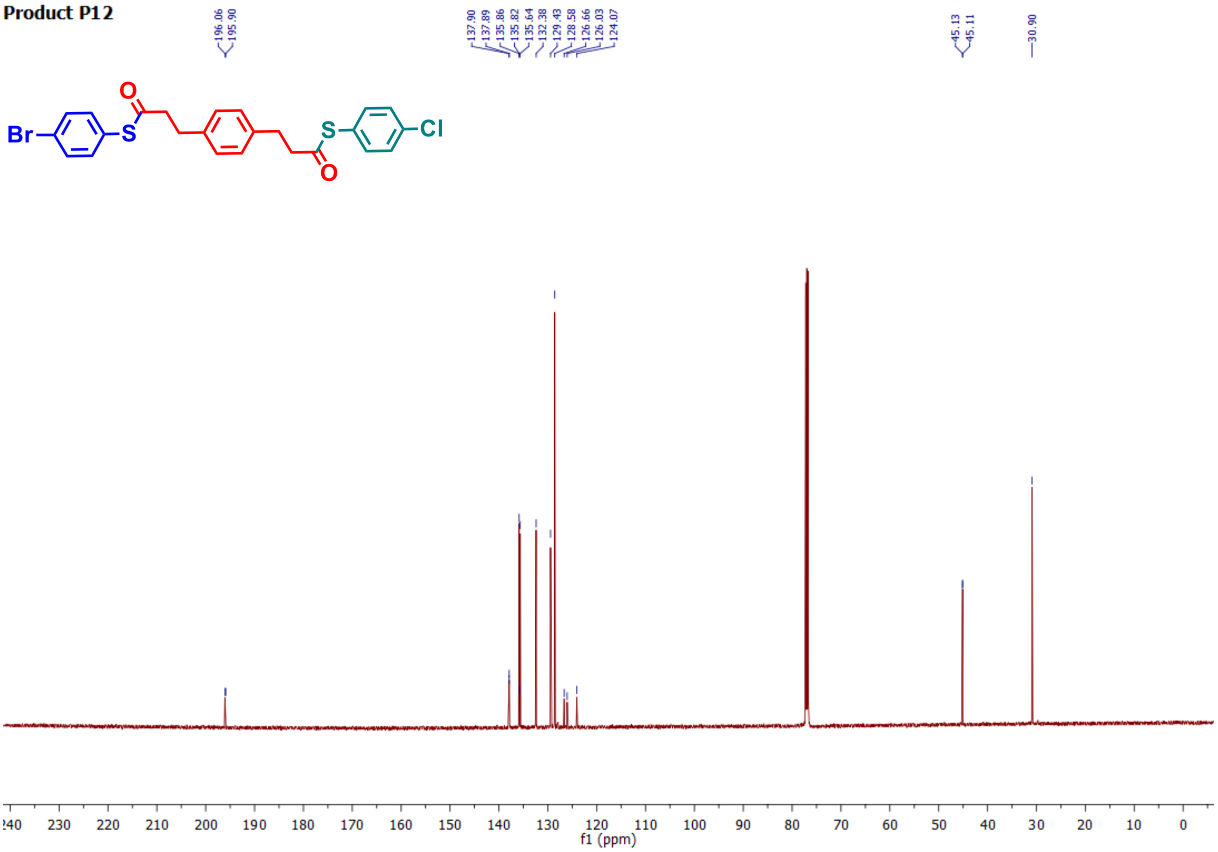

Figure S25. ^{13}C NMR (101 MHz, $CDCl_3$) of product **P12**

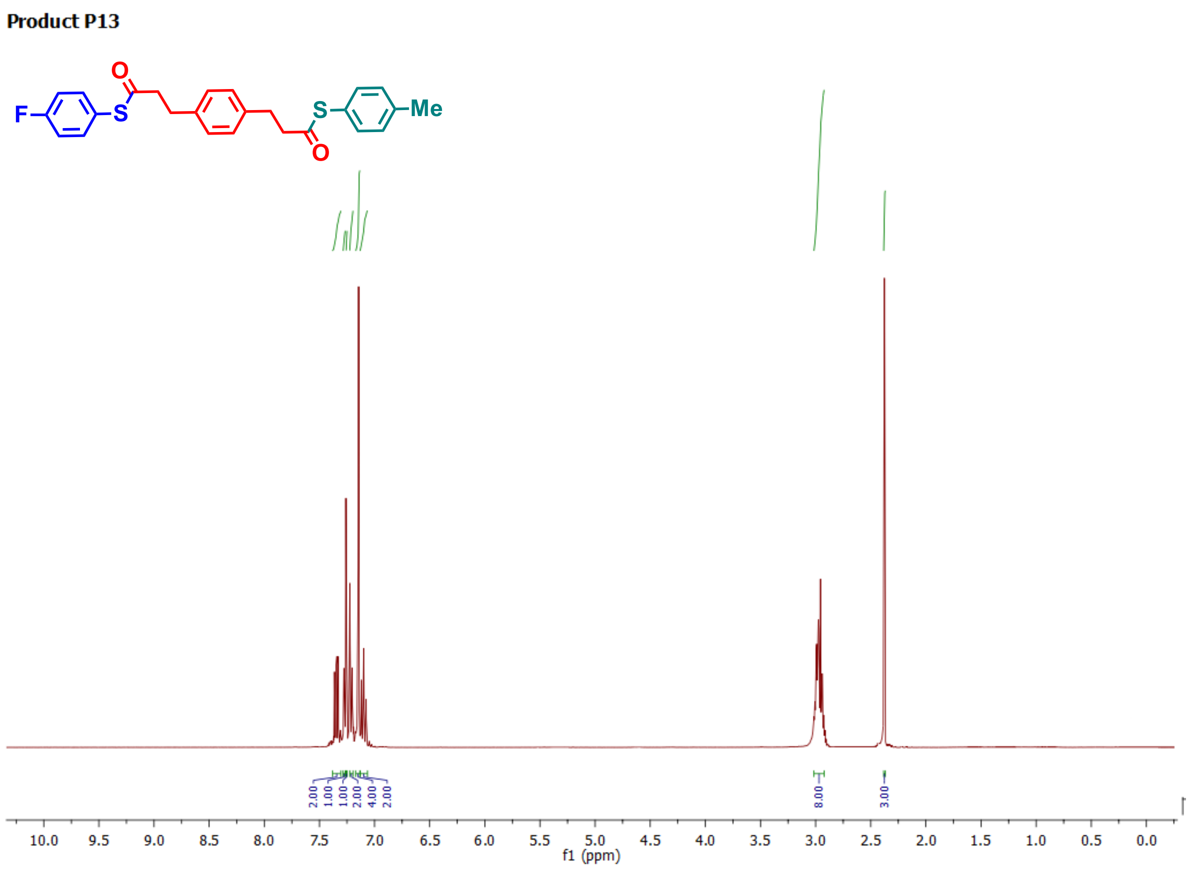

Figure S26. ^{1}H NMR (400 MHz, $CDCl_3$) of product **P13**

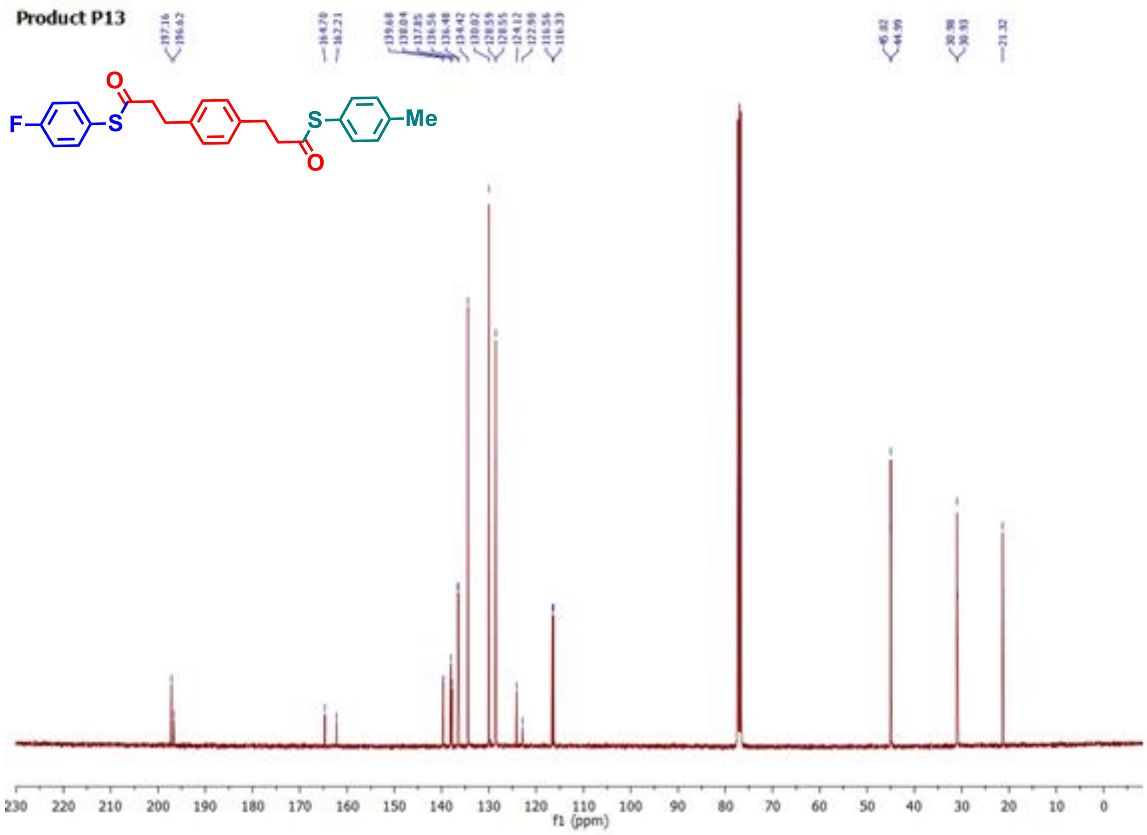

Figure S27. ^{13}C NMR (101 MHz, $CDCl_3$) of product **P13**

Product P14

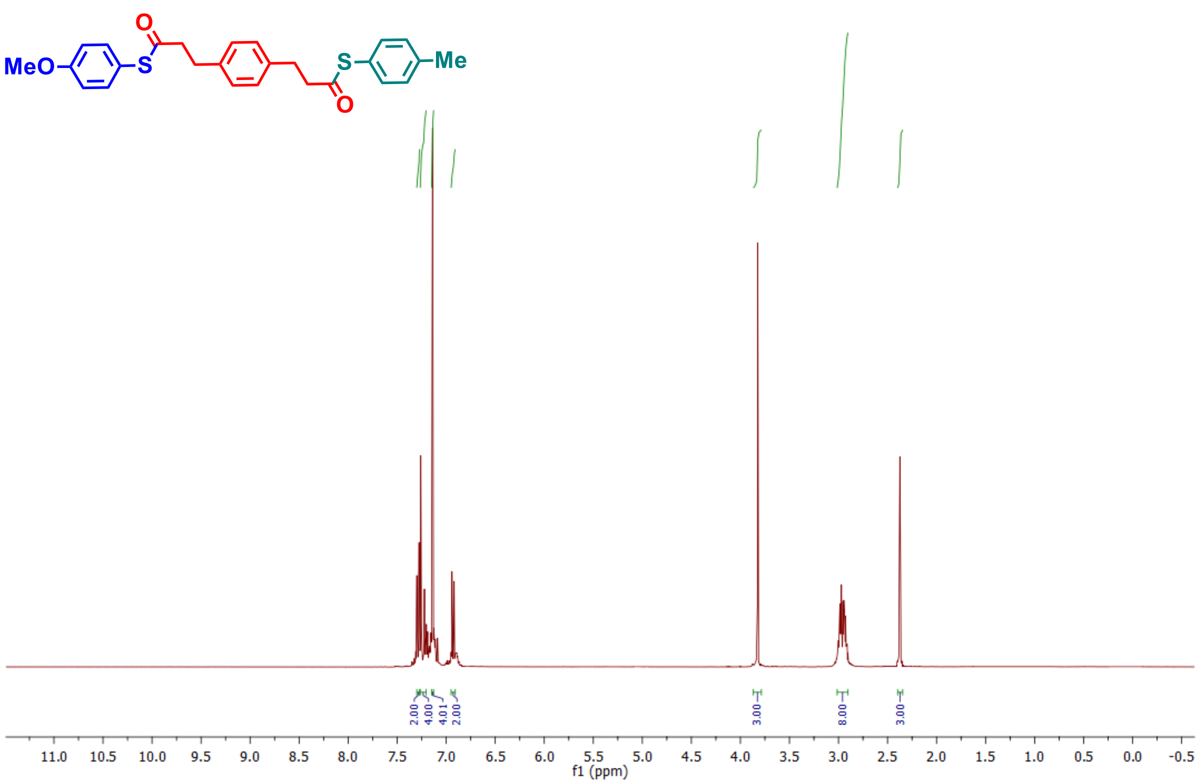

Figure S28. ^{1}H NMR (400 MHz, $CDCl_3$) of product **P14**

Product P14

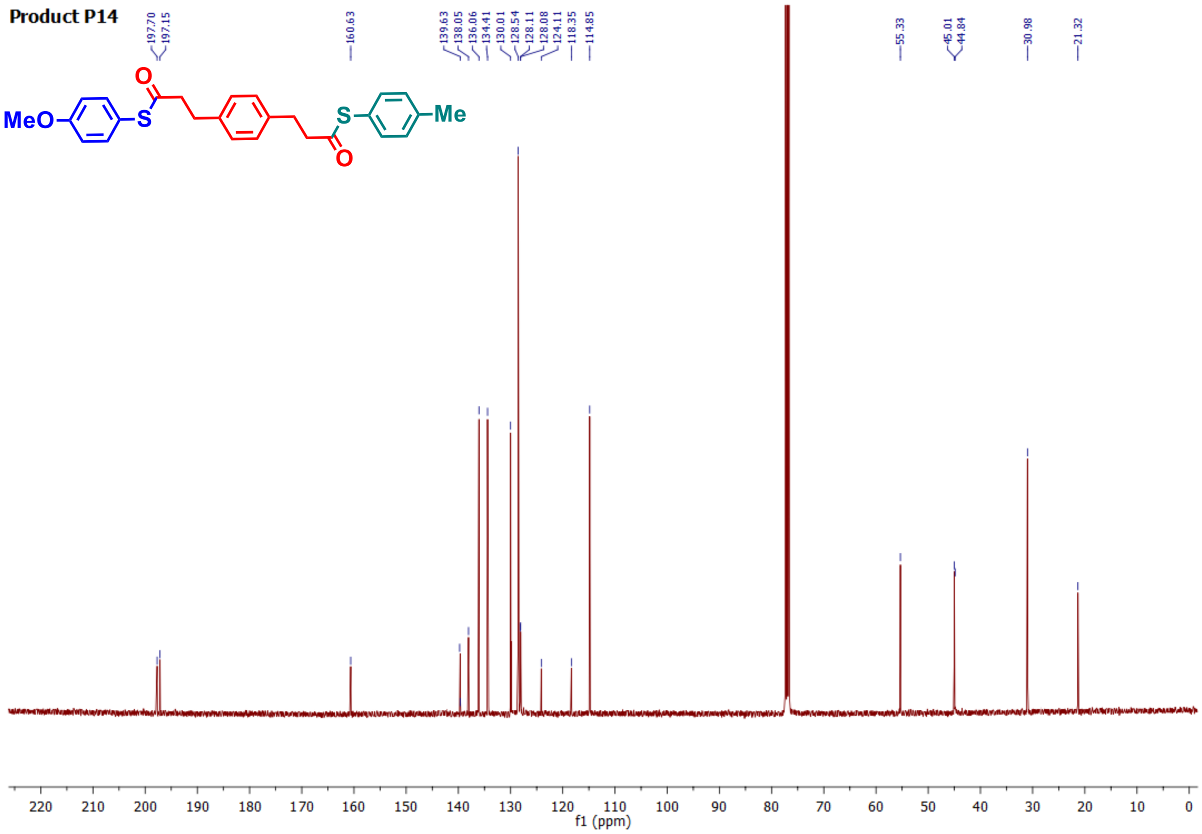

Figure S29. ^{13}C NMR (101 MHz, $CDCl_3$) of product **P14**

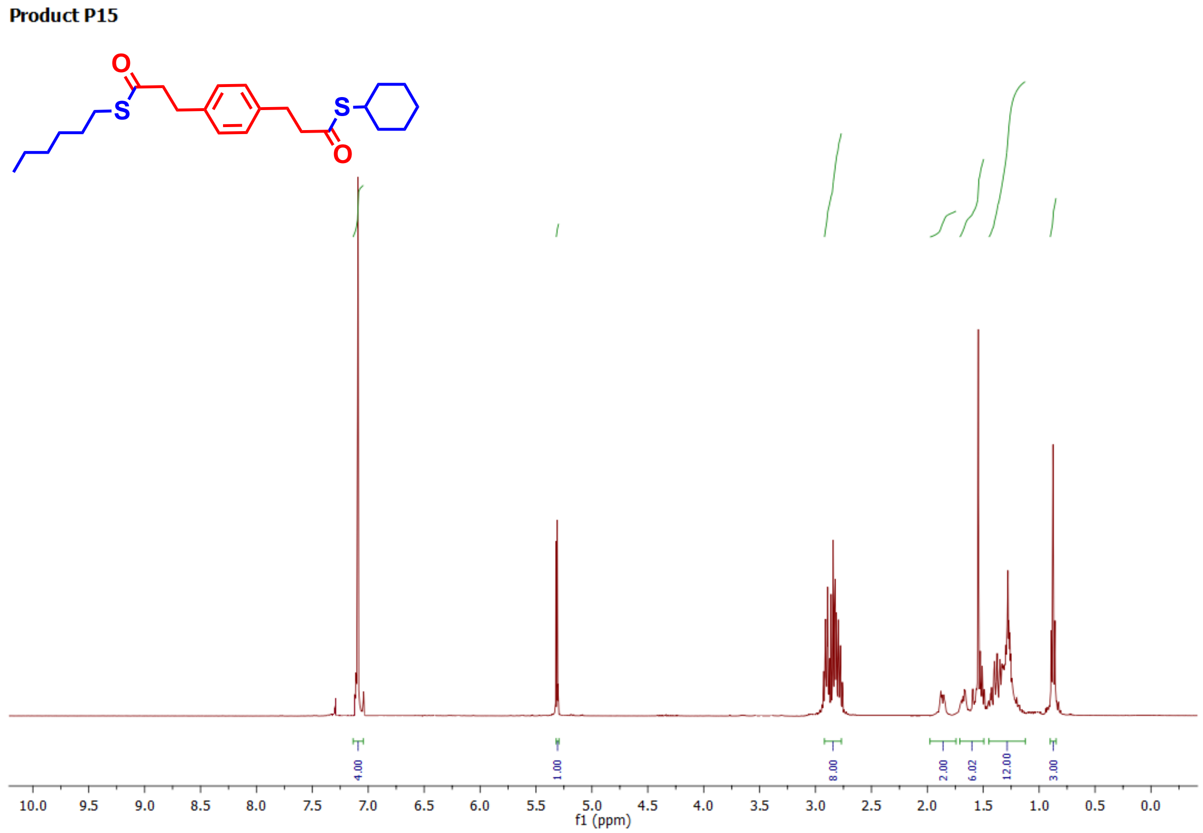

Figure S30. ^{1}H NMR (400 MHz, $CDCl_3$) of product **P15**

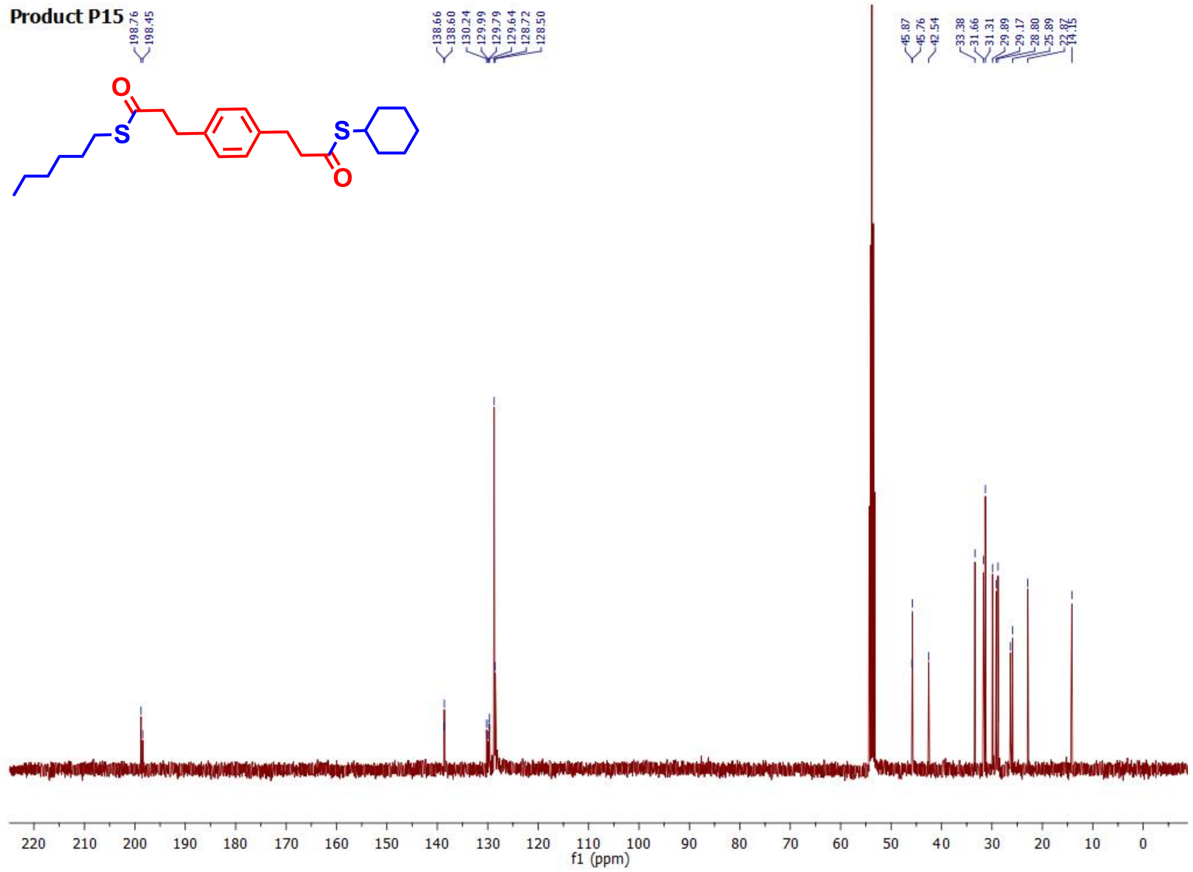

Figure S31. ^{13}C NMR (101 MHz, $CDCl_3$) of product **P15**

3. XRD analysis

3.1. X-ray crystallography of the products P1, P2, P3, P5

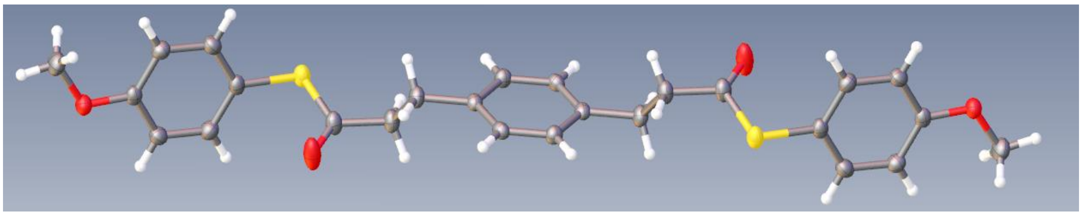

PRODUCT P1

Figure S32. A perspective views of the molecule **P1**

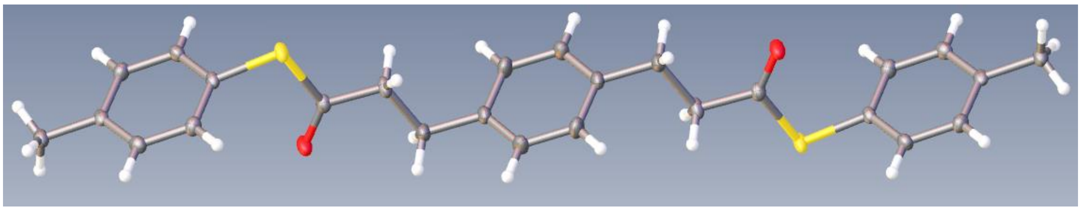

PRODUCT P2

Figure S33. A perspective views of the molecule **P2**

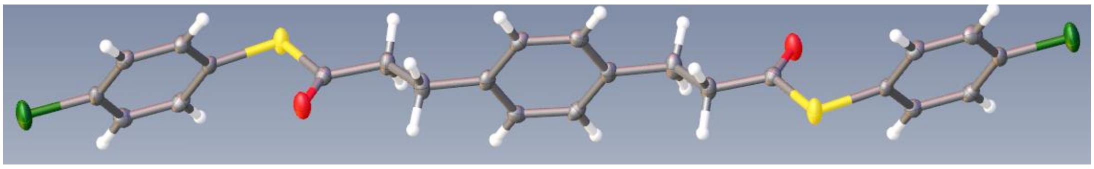

PRODUCT P3

Figure S34. A perspective views of the molecule **P3**

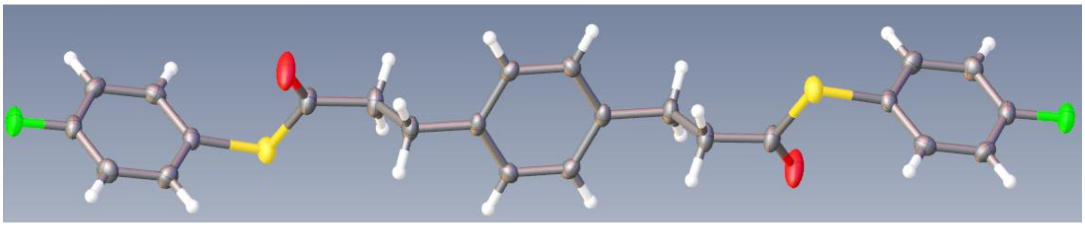

PRODUCT P5

Figure S35. A perspective views of the molecule **P5**

A colourless, block-shaped crystal was mounted on the goniometer. Data for **P1, P2, P3 and P5** were collected from a single crystal at 100 K on a Bruker D8 QUEST KAPPA diffractometer with a microfocus sealed tube using a multilayer mirror as monochromator and a Bruker

PHOTON III CPAD detector. The diffractometer was equipped with an Oxford Cryostream 600 low temperature device and used Mo K_α radiation (λ = 0.71073 Å). All data for **P1** were integrated with SAINT V8.41, yielding 16284 reflections of which 2122 where independent (average redundancy 7.67) and 93.3% were greater than 2σ(F^2).[1] All data for **P2** were integrated with SAINT V8.41, yielding 9724 reflections of which 1985 where independent (average redundancy 4.90) and 83.4% were greater than 2σ(F^2). [1] All data for **P3** were integrated with SAINT V8.41, yielding 17518 reflections of which 2022 where independent (average redundancy 8.66) and 91.0% were greater than 2σ(F^2).[1] A Multi-Scan absorption correction using SADABS 2016/2 was applied.[2] All data for **P5** were integrated with SAINT V8.41, yielding 13355 reflections of which 1944 where independent (average redundancy 6.87) and 92.7% were greater than 2σ(F^2). [1] A Multi-Scan absorption correction using SADABS 2016/2 was applied.[2] The structure was solved by dual methods with SHELXT 2018/2 and refined by full-matrix least-squares methods against F^2 using XL.[3,4] All non-hydrogen atoms were refined with anisotropic displacement parameters. All hydrogen atoms were refined isotropic on calculated positions using a riding model with their U_{iso} values constrained to 1.5 times the U_{eq} of their pivot atoms for terminal sp^3 carbon atoms and 1.2 times for all other carbon atoms. Crystallographic data for the structures reported in this paper have been deposited with the Cambridge Crystallographic Data Centre.[5] CCDC 2494401, CCDC 2494402, CCDC 2529205, CCDC 2494403 contain the supplementary crystallographic data for this paper. These data can be obtained free of charge from The Cambridge Crystallographic Data Centre via www.ccdc.cam.ac.uk/structures. This report and the CIF file were generated using FinalCif.[6]

Table S1. Crystal data and structure refinement for **P1-P3 and P5.**

	**Product P1**	**Product P2**	**Product P3**	**Product P5**
Empirical formula	$C_{26}H_{26}O_4S_2$	$C_{26}H_{23}O_2S_2$	$C_{24}H_{20}Cl_2O_2S_2$	$C_{24}H_{20}F_2O_2S_2$
Formula weight	466.59	431.59	475.45	442.54
Temperature [K]	100	100	100	100
Crystal system	triclinic	monoclinic	orthorhombic	monoclinic
Space group (number)	$P\overline{1}$ (2)	$P2_1/n$ (14)	$Pbca$ (61)	$P2_1/n$ (14)
a [Å]	8.2747(8)	5.8438(6)	17.0622(15)	9.3025(13)
b [Å]	8.6071(8)	5.1101(6)	7.5048(7)	12.0965(17)
c [Å]	9.7371(9)	36.409(4)	17.1536(14)	10.3564(16)
α [°]	68.372(3)	90	90	90
β [°]	68.165(3)	90.541(4)	90	115.404(4)
γ [°]	68.460(3)	90	90	90
Volume [Å^3]	576.75(10)	1087.2(2)	2196.5(3)	1052.7(3)
Z	1	2.0	4.0	2.0
ρ_{calc} [gcm^{-3}]	1.343	1.328	1.438	1.396
μ [mm^{-1}]	0.262	0.266	4.589	0.289
F(000)	246	460	984	460
Crystal size [mm^3]	0.1×0.3×0.5	0.05×0.3×0.4	0.05×0.2×0.4	0.1×0.4×0.6
Crystal colour	colourless	colourless	colourless	colourless
Crystal shape	block	plate	plate	plate

Radiation	Mo K_α (λ=0.71073 Å)	Mo K_α (λ=0.71073 Å)	Cu K_α (λ=1.54178 Å)	Mo K_α (λ=0.71073 Å)
2θ range [°]	4.68 to 50.81 (0.83 Å)	4.48 to 50.95 (0.83 Å)	10.31 to 136.89 (0.83 Å)	4.93 to 50.98 (0.83 Å)
Index ranges	$-9 \le h \le 9$<br>$-10 \le k \le 10$<br>$-11 \le l \le 11$	$-7 \le h \le 6$<br>$-5 \le k \le 6$<br>$-43 \le l \le 43$	$-19 \le h \le 20$<br>$-7 \le k \le 9$<br>$-20 \le l \le 20$	$-11 \le h \le 11$<br>$-14 \le k \le 13$<br>$-12 \le l \le 12$
Reflections collected	16284	9724	17518	13355
Independent reflections	2122<br>R_{int} = 0.0386<br>R_{sigma} = 0.0261	1985<br>R_{int} = 0.0545<br>R_{sigma} = 0.0425	2022<br>R_{int} = 0.0403<br>R_{sigma} = 0.0230	1944<br>R_{int} = 0.0439<br>R_{sigma} = 0.0276
Completeness to θ = 25.242°	99.6	99.3	99.9	99.8
Data / Restraints / Parameters	2122 / 0 / 146	1985 / 0 / 137	2022 / 0 / 136	1944 / 0 / 136
Goodness-of-fit on F^2	1.033	1.047	1.091	1.059
Final R indexes [$I \geq 2\sigma(I)$]	R_1 = 0.0327<br>wR_2 = 0.0786	R_1 = 0.0377<br>wR_2 = 0.0899	R_1 = 0.0343<br>wR_2 = 0.0851	R_1 = 0.0342<br>wR_2 = 0.0855
Final R indexes [all data]	R_1 = 0.0352<br>wR_2 = 0.0804	R_1 = 0.0584<br>wR_2 = 0.0979	R_1 = 0.0385<br>wR_2 = 0.0886	R_1 = 0.0375<br>wR_2 = 0.0883
Largest peak/hole [eÅ^{-3}]	0.49/−0.31	0.27/−0.31	0.35/−0.38	0.33/−0.28

4. FT-IR analysis

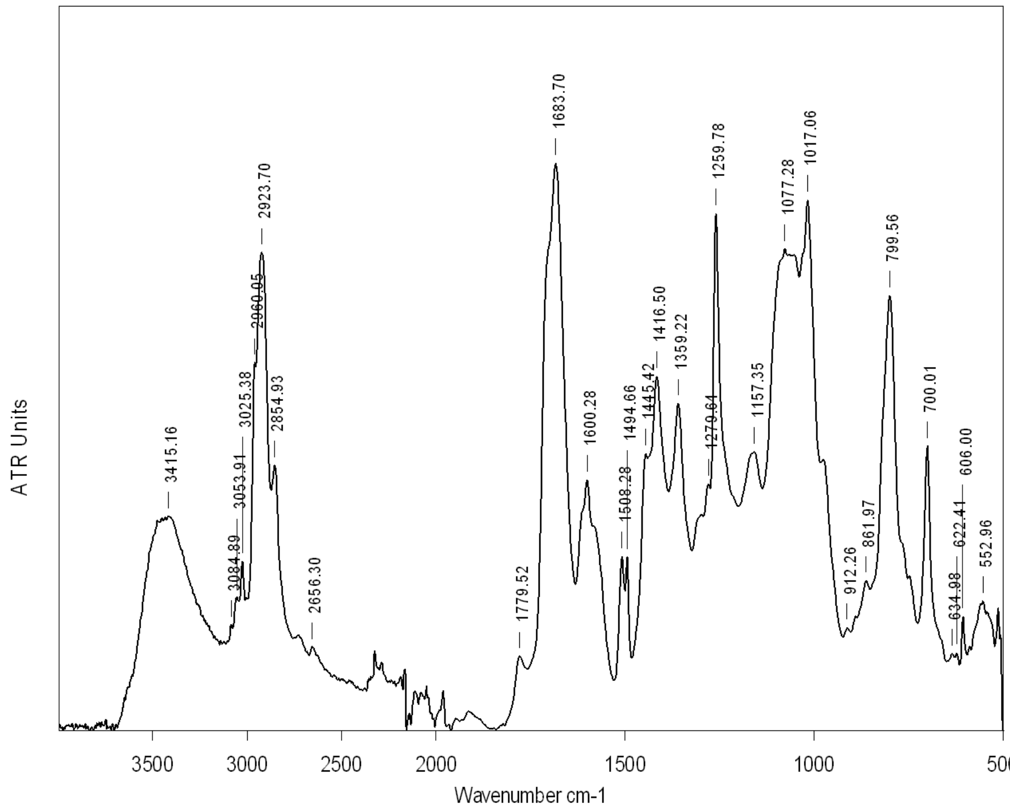

Figure S36. FT-IR spectrum of product **P16**

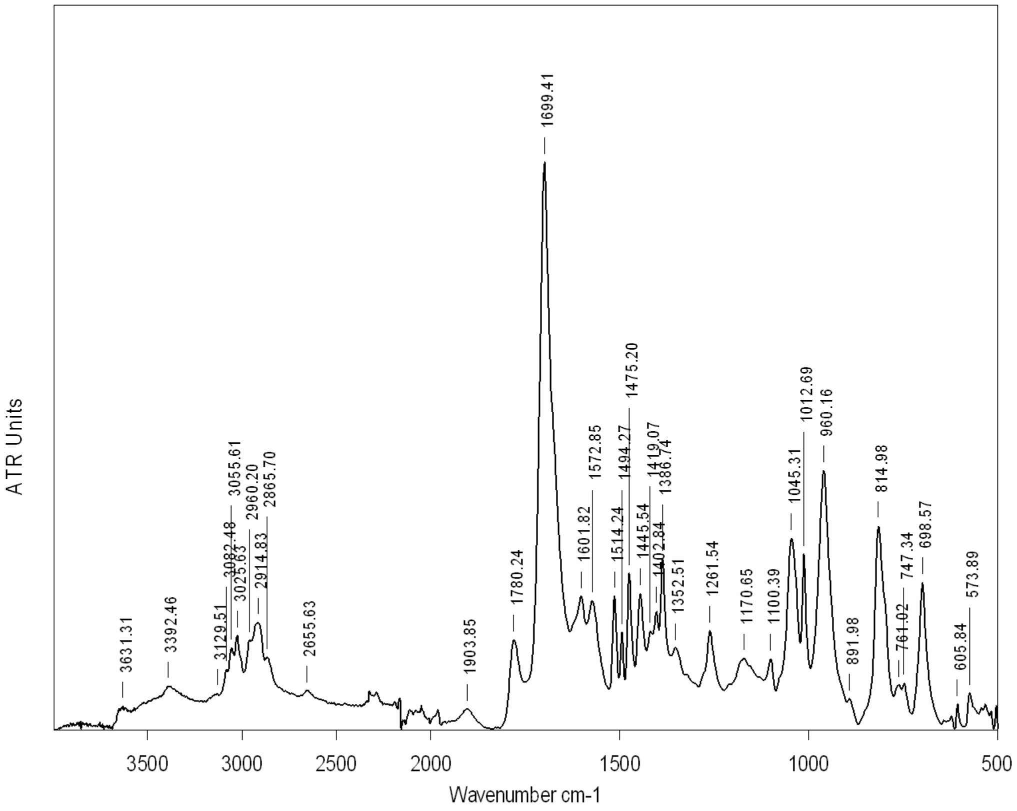

Figure S37. FT-IR spectrum of product **P17**

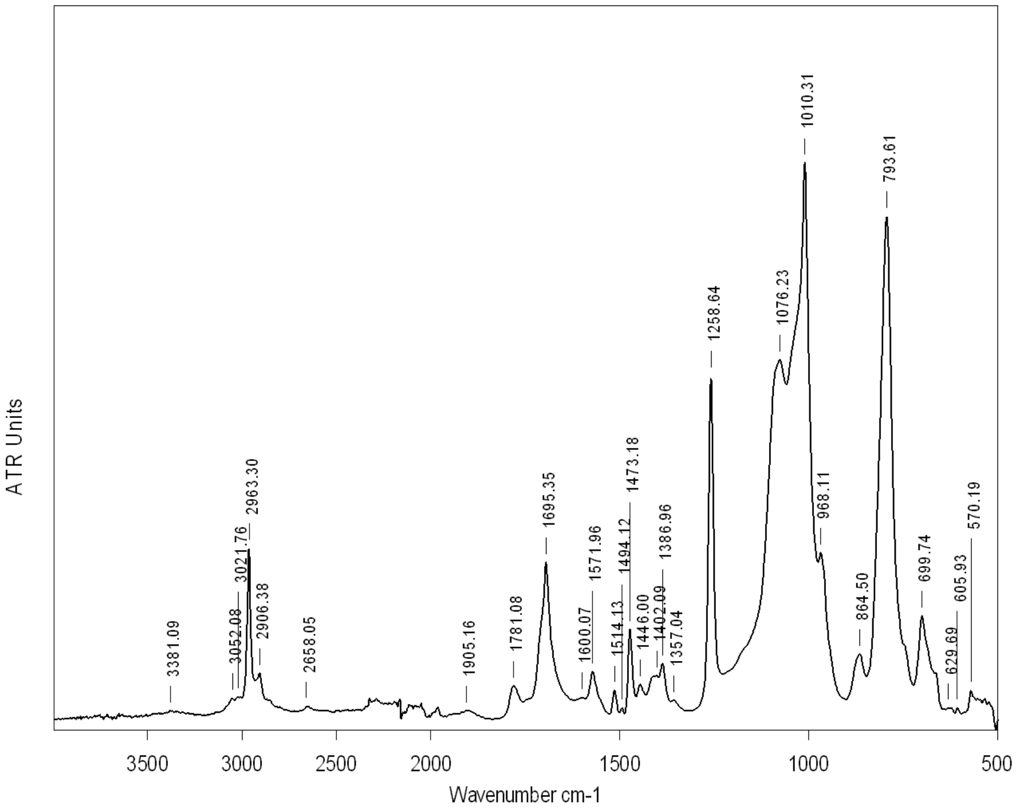

Figure S38. FT-IR spectrum of product **P18**

5. DSC Analysis

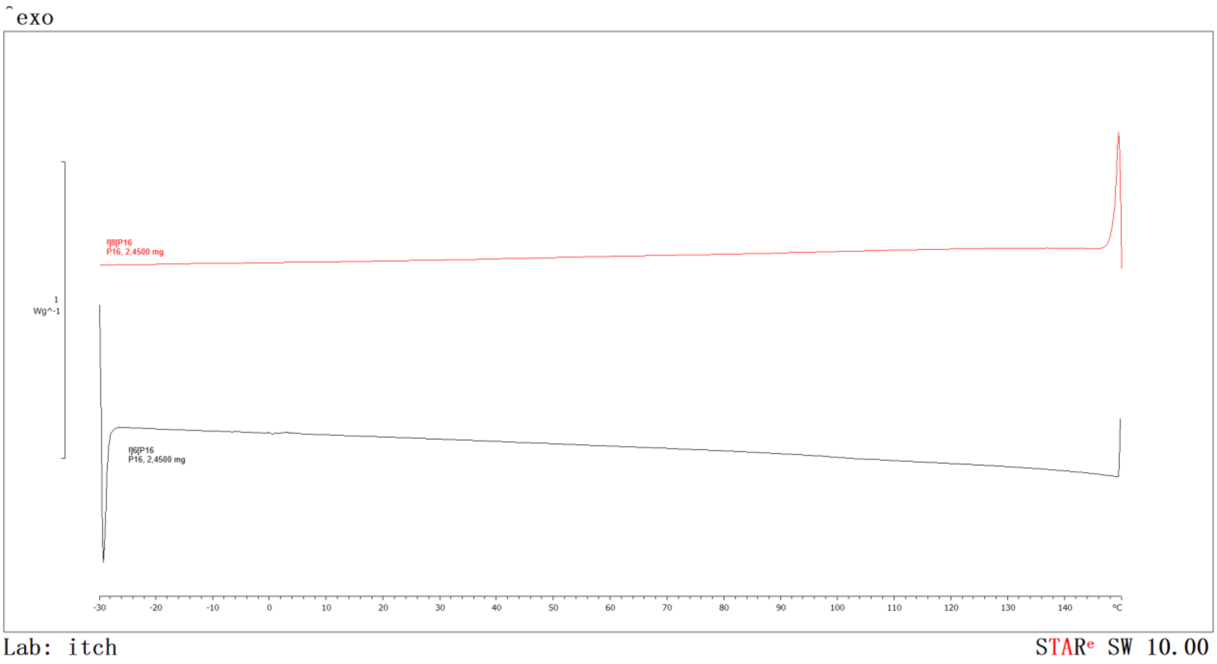

Figure S39. Heat flow curves for the first and second heating-cooling runs for sample **P16**

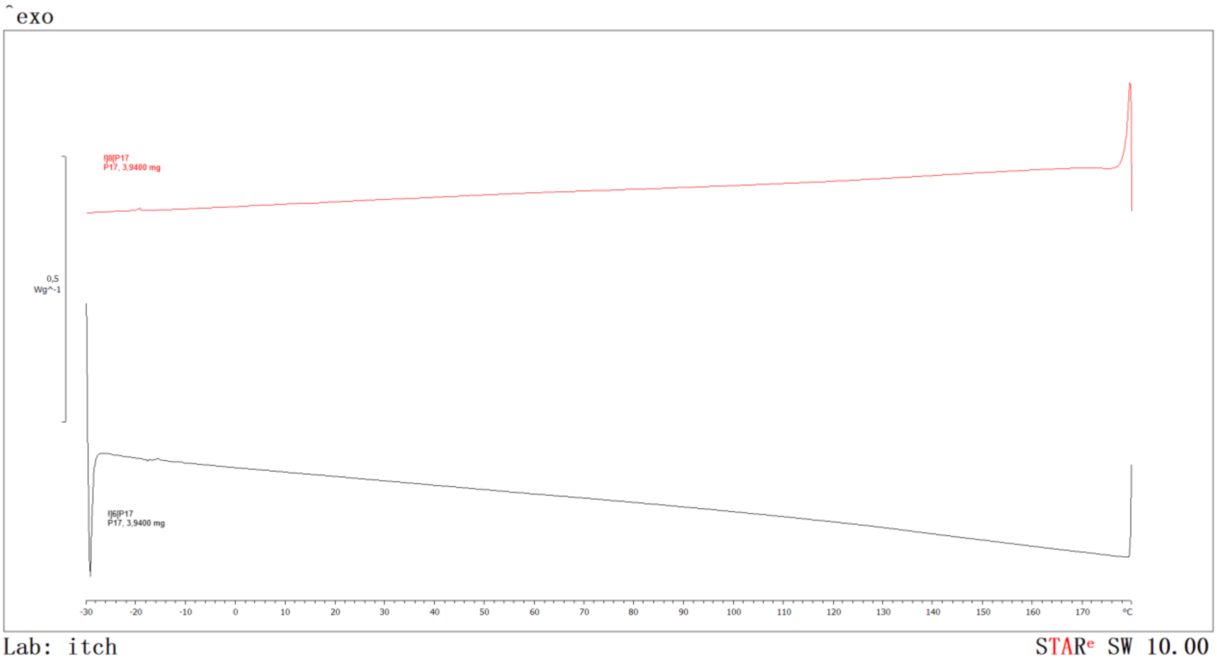

Figure S40. Heat flow curves for the first and second heating-cooling runs for sample **P17**

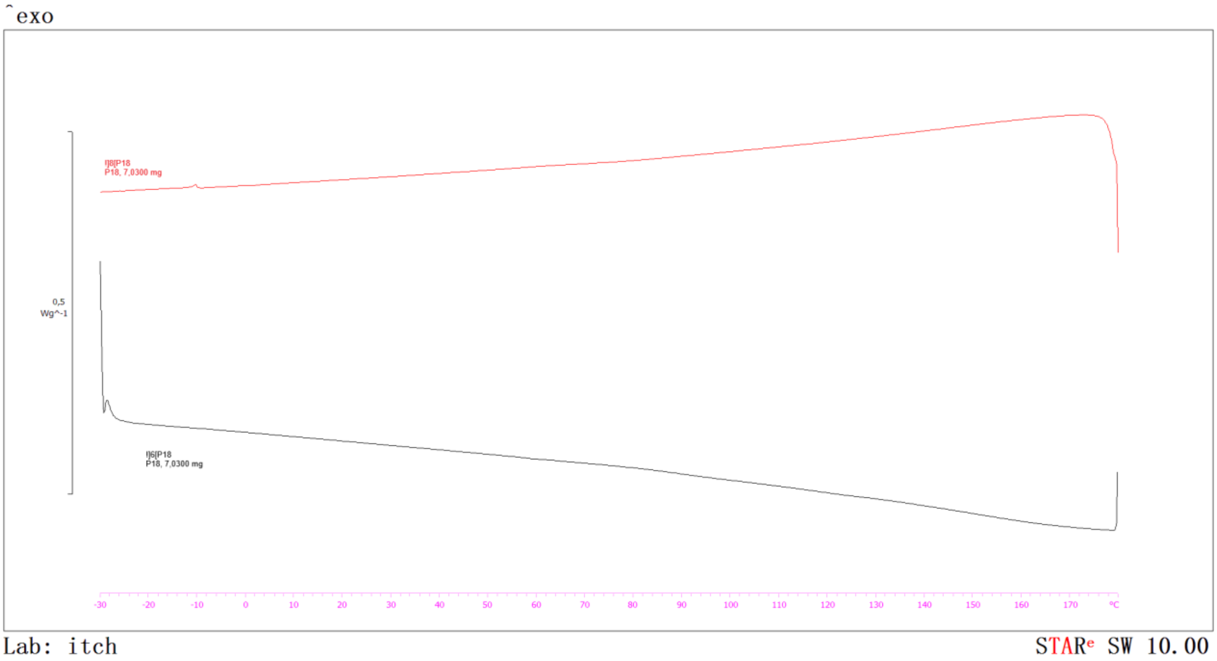

Figure S41. Heat flow curves for the first and second heating-cooling runs for sample **P18**

6. Statistical analysis

The reaction of (2E,2'E)-3,3'-(1,4-phenylene)diacrylaldehyde (**1a**) and 4-methoxythiophenol (**2a**) was repeated five times under optimized conditions.

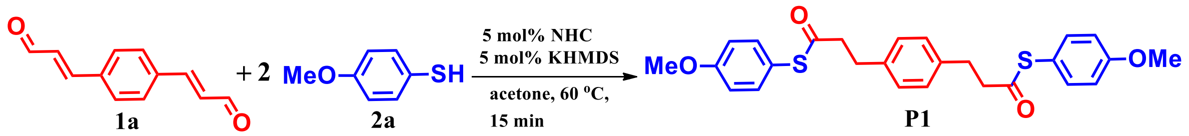

Entry	Yield of P1 [%][a]
1	94
2	93
3	90
4	92
5	93

Reaction conditions: [**1a**]:[**2a**] = 1:2, [**NHC**]:[**KHMDS**] = 1:1, argon.

[a] Determined by ^{1}H NMR of post-reaction mixture.

For the obtained yield results, the standard deviation was calculated (SD=1.52) and a Gaussian curve illustrating the reproducibility of yields and reaction conditions was created.

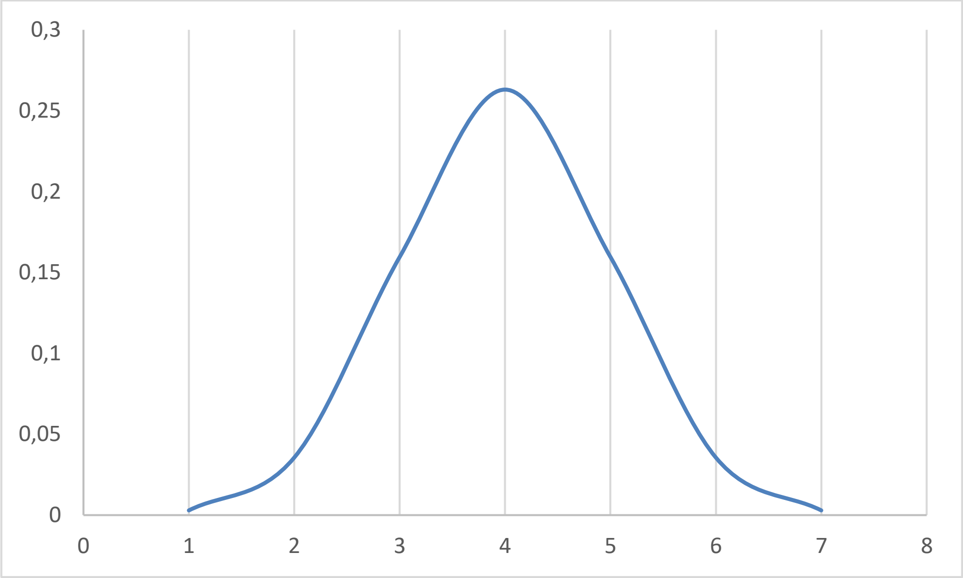

Figure S42. Normalized Gaussian curve presented reproducibility of yields and reaction conditions.

7. References

[1] Bruker (2012). *SAINT*, Version 8.41. Bruker AXS Inc., Madison, Wisconsin, USA. Available online: Bruker AXS official website (accessed on 1 July 2026).

[2] L. Krause, R. Herbst-Irmer, G. M. Sheldrick, D. Stalke, *J. Appl. Cryst.* **2015**, *48*, 3–10, doi:10.1107/S1600576714022985.

[3] G. M. Sheldrick, *Acta Cryst.* **2015**, *A71*, 3–8, doi:10.1107/S2053273314026370.

[4] G. M. Sheldrick, *Acta Cryst.* **2015**, *C71*, 3–8, doi:10.1107/S2053229614024218.

[5] C. R. Groom, I. J. Bruno, M. P. Lightfoot, S. C. Ward, *Acta Cryst.* **2016**, *B72*, 171–179, doi:10.1107/S2052520616003954.

[6] Kratzert, D. FinalCif, (Bruker Edition). Available online: https://dkrat ert.de/finalcif.html (accessed on 23 June 2026).
